# Assessing the extent to which front-of-pack labelling regulations could support healthy eating among Canadians

**DOI:** 10.1371/journal.pone.0330720

**Published:** 2025-10-08

**Authors:** Jennifer J. Lee, Christine Mulligan, Hayun Jeong, Mary R. L’Abbe

**Affiliations:** 1 Department of Nutritional Sciences, Temerty Faculty of Medicine, University of Toronto, Toronto, Ontario, Canada; 2 School of Nutrition, Faculty of Community Services, Toronto Metropolitan University, Toronto, Ontario, Canada; Tabba Heart Institute, PAKISTAN

## Abstract

Canada mandated front-of-pack labelling (FOPL) regulations, requiring pre-packaged foods meeting and/or exceeding thresholds for nutrients-of-concern (saturated fat, sugars, sodium) to display a ‘High in’ nutrition symbol. Although FOPL regulations align with one of the recommendations of Canada’s food guide (CFG), there is limited evidence on how well the regulations could support healthy eating among Canadians. The objective of this study was to evaluate the Canadian pre-packaged food supply according to FOPL regulations and to assess the extent to which the regulations could support healthy eating among Canadians using the Canadian Food Scoring System (CFSS), a nutrient profile model based on the recommendations of CFG. Using a branded food composition database (n = 17,008), pre-packaged foods were categorized according to FOPL regulations and the CFSS. According to FOPL regulations, approximately 54% of pre-packaged foods would display a ‘High in’ nutrition symbol for meeting and/or exceeding thresholds for at least one nutrient-of-concern. According to the CFSS, approximately 53% of foods were a ‘poor’ or ‘very poor’ choice, while 25% were a ‘good’ or ‘excellent’ choice. Foods that would not display a ‘High in’ nutrition symbol showed significant variation in their healthfulness, with 45% containing low amounts of nutritious foods recommended by CFG. Our findings highlight that many pre-packaged foods in Canada do not represent healthy choices. Although many of these foods will be highlighted with a ‘High in’ nutrition symbol when FOPL regulations are implemented, many foods that would not display a ‘High in’ nutrition symbol do not align well with the recommendations of CFG, particularly those with a variety of multiple ingredients (e.g., many breads, breakfast cereals, combination dishes). Additional tools and strategies are required to support Canadians make healthy food choices.

## Introduction

Poor diet is one of the main risk factors for noncommunicable disease (NCD) morbidity and mortality worldwide and in Canada [1,2]. Public health policies promoting healthy diets have been used to help curtail the rising incidence of NCDs. Canada introduced the *Healthy Eating Strategy* in 2016 to improve the food environment and diets of Canadians by ‘making the healthier choice, the easier choice [3].’ As part of the *Healthy Eating Strategy*, several food and nutrition policies were introduced, including the publication of front-of-pack labelling (FOPL) regulations and updating Canada’s food guide (CFG). Canadian FOPL regulations mandate pre-packaged food and beverage products (“foods” hereinafter) meeting and/or exceeding thresholds for nutrients-of-concern (i.e., saturated, total sugars, and sodium) to display a ‘High in’ nutrition symbol on the front of the food package by January, 2026 [4]. A previous study examining the potential impact of proposed FOPL regulations (pre-published in 2018 [5]) on the Canadian pre-packaged food supply showed that 64% of pre-packaged foods would be required to display a ‘High in’ nutrition symbol [6]. However, the previous analysis used the proposed regulations, and no study has examined the potential impact on the pre-packaged food supply since the publication of the final FOPL regulations in 2022.

In 2019, CFG was updated to provide the most up-to-date, evidence based dietary recommendations for Canadians over the age of 2 years to promote healthy eating, support overall nutritional well-being, and reduce the risk of nutrition-related NCDs [7]. Canada’s Dietary Guidelines for Health Professionals and Policymakers (CDG) was also published to provide more detailed dietary guidelines and their scientific rationale [14]. There are three main Guidelines in CDG [14]: (1) “Nutritious foods are the foundation for healthy eating;” (2) “Processed or prepared foods and beverages that contribute to excess sodium, free sugars, or saturated fat undermine healthy eating and should not be consumed regularly;” and (3) “Food skills are needed to navigate the complex food environment and support healthy eating.” Although these recommendations are qualitative in nature, regulatory definitions of foods from labelling standards and regulations can be used to set quantitative criteria (e.g., existing nutrient content claim criteria to differentiate lean vs. non-lean meat; ‘high in’ nutrients-of-concern for foods that would display a ‘High in’ nutrition symbol according to FOPL regulations) to operationalize the recommendations, specifically Guidelines 1 and 2, which focus on individual food choices. For instance, Guideline 2 of CDG will be operationalized with the implementation of FOPL regulations to highlight foods ‘high in’ nutrient(s)-of-concern. However, it is unclear how well FOPL regulations could support adherence to other recommendations of CFG and Guideline 1 of CDG, which include a variety of recommendations on nutritious foods for healthy eating beyond foods to limit based on nutrient levels. Further, the application of nutritious food recommendations may be particularly challenging for pre-packaged foods subject to FOPL regulations, as they inherently undergo some degree of processing, contain varying nutrient levels (e.g., high levels of sodium and fibre, or low levels of sugars but high levels of saturated fat), and combinations of ingredients, some of which may conflict with the nutritious food recommendations (e.g., combination of whole grains and high sugar levels, or beans and high salt levels). Therefore, the objectives of this study were (i) to examine the pre-packaged food supply according to final FOPL regulations, and (ii) to assess the extent to which FOPL regulations could support healthy eating in line with the recommendations of CFG and CDG.

## Materials and methods

### Branded food composition database – Food Label Information and Price (FLIP)

The Food Label Information and Price (FLIP), a nationally-representative, branded food composition database, was used in this study. FLIP 2017 data were collected between May and September 2017 from top Canadian food retailers (Loblaws, Sobeys, and Metro), representing over 60% of the grocery retail market share at the time of data collection [8,9]. FLIP 2017 contains nutritional information for 17,671 unique pre-packaged foods, including product name, company/brand, Nutrition Facts table (NFt) information, ingredients list, universal product code, and photos of all sides of the package. Foods with missing ingredient information and foods not regulated by FOPL regulations (e.g., foods for special dietary use, non-caloric sweetening agents) were removed from the analysis (n = 663). Although foods for <1-year-olds would be exempted from FOPL regulations, all foods for <4-year-olds were included in the analysis, as only the minimum age for consumption (e.g., ≥ 6-month-olds), not maximum age for consumption, were indicated in these foods. The final sample size was 17,008. All foods were classified according to Health Canada’s Table of Reference Amounts for Food (TRA) categories, which is used to determine the common use of a food and the serving size for foods on the NFt [10].

### Front-of-pack labelling (FOPL) regulations

All foods in FLIP 2017 were evaluated according to the final Canadian FOPL regulations, published in 2022 [4]. Foods were first assessed against three types of exemption criteria [4] and identified as “Exempted from FOPL.” The exemption criteria were: (i) health-related exemptions for foods that have been shown health benefits (e.g., fruits and vegetables, oils high in unsaturated fats); (ii) technical-related exemptions for foods that are not required to display a NFt (e.g., raw single ingredient meats); and (iii) practical-related exemptions for foods that are well-known sources of nutrients-of-concern (e.g., honey, butter, table salt). The remaining foods were assessed for their levels of three nutrients-of-concern: saturated fat, total sugars, and sodium. The threshold levels were set based on the percent daily values (%DV) per reference amount reported in TRA [10] and age groups for each nutrient [4]. Each food’s levels of nutrients-of-concern per reference amount as reported in TRA (or the manufacturer-suggested serving size as reported on NFt, whichever is larger) were compared against the thresholds. Foods that required reconstitution with water or any other liquids (e.g., powdered drink mixes, bouillon cubes) were assessed based on the reference amount of its prepared form [4]. Foods were then categorized into one of five categories: (i) exempted from FOPL; (ii) <thresholds for all three nutrients-of-concern; (iii) would display a ‘High in’ nutrition symbol for one nutrient-of-concern; (iv) would display a ‘High in’ nutrition symbol for two nutrients-of-concern; and (v) would display a ‘High in’ nutrition symbol for three nutrients-of-concern. Foods that would display a ‘High in’ nutrition symbol were further sub-categorized by the type of ‘high in’ nutrient-of-concern to examine the prevalence of each ‘high in’ nutrient-of-concern.

### Canadian Food Scoring System (CFSS)

To examine the extent to which FOPL regulations could support healthy eating among Canadians, the Canadian Food Scoring System (CFSS) was applied to all products in FLIP 2017. The CFSS is a nutrient profile model developed based on the recommendations of CFG and its dietary guidelines using existing Canadian labelling regulations (e.g., FOPL) and standards (e.g., TRA) to set quantitative criteria for the recommendations in CFG, described in detail elsewhere [11]. Briefly, there were three main steps of the CFSS.

The first step was the categorization and assignment of points to foods according to the nutritious food point system, which reflected the recommendations on nutritious foods in CFG and Guideline 1 of CDG: “Nutritious foods are the foundation for healthy eating [12].” Foods (single-ingredient vs. multi-ingredient foods based on the number of ingredients) and beverages were categorized and awarded points (range: 0–100) based on their alignment with the nutritious food recommendations of CFG and Guideline 1 of CDG (e.g., “Eat plant-based protein foods more often” for the protein food recommendations).

The second step was the application of a deduction proportion using FOPL regulations, which represented the recommendations on nutrients-of-concern in CFG and Guideline 2 of CDG: “Processed or prepared foods and beverages that contribute to excess sodium, free sugars, or saturated fat undermine healthy eating and should not be consumed regularly [12].” Foods were assessed against the exemption criteria and thresholds of Canadian FOPL regulations [4] to identify if and how many nutrients-of-concern (i.e., saturated fat, total sugars, sodium) a food would be indicated as having ‘high in’ levels of these nutrients with a ‘High in’ nutrition symbol. The presence and the number of ‘High in’ nutrients-of-concern a product would need to display (i.e., ≥ thresholds) was used to assign the deduction proportion. Foods that met the exemption criteria or had nutrient levels below thresholds (i.e., would not display a ‘High in’ nutrition symbol) were not assigned any deductions, while there was a 50% deduction for foods meeting and/or exceeding thresholds for 1 nutrient; 65% for 2 nutrients, and 80% for all 3 nutrients.

The third step comprised of calculating the final score (range:10–100) by adding up the points from Steps 1 and 2 to categorize foods into one of five categories: ‘very poor (CFSS score range: 10-29),’ ‘poor (30-49),’ ‘fair (50-69),’ ‘good (70-89),’ or ‘excellent (90-100)’ choice according to the recommendations of CFG and CDG [11].

### Comparing FOPL regulations with the recommendations of CFG

Since the CFSS operationalized the recommendations on nutrient-of-concern using FOPL regulations, the CFSS is inherently entangled with the regulations. Therefore, to determine the extent to which FOPL regulations could support adherence to CFG, the nutritious food points from Step 1 of the CFSS were examined to assess the presence of nutritious foods recommended by CFG and Guideline 1 of CDG, independent of FOPL regulations (i.e., Step 2 of the CFSS). The nutritious food points were presented as means and standard deviations (SD) and as proportions by quintile groups of points (i.e., 0–20; 21–40; 41–60; 61–80; 81–100). The nutritious food points of 0–40 (i.e., the first two quintiles) were considered to have low amounts of nutritious foods recommended by CFG, while 61–100 points (i.e., the last two quintiles) were considered to have high amounts of nutritious foods. The nutritious food points from Step 1 of the CFSS and the final CFSS scores were also examined based on the 5-level FOPL regulation categories (i.e., exempted from regulations; display no ‘High in’ nutrition symbol for having below thresholds for all three nutrients-of-concern; display a ‘High in’ nutrition symbol for one nutrient-of-concern; display a ‘High in’ nutrition symbol for two nutrients-of-concern; and display a ‘High in’ nutrition symbol for three nutrients-of-concern). The differences in the Step 1 nutritious food points and the final CFSS scores across all FOPL regulation categories were examined using a one-factor analysis of variance (ANOVA), adjusted for Tukey-Kramer’s *post hoc* analyses overall and by TRA category. To further examine the discrepancies between FOPL regulations and the CFSS, the number and proportion of foods that would not display a ‘High in’ nutrition symbol were examined by the CFSS categories. All analyses were performed using SAS (version 9.4, SAS Institute Inc., Cary, NC, USA). Statistical significance was set at p < 0.05.

## Results

### Pre-packaged foods categorized according to FOPL regulations

**Fig 1:**
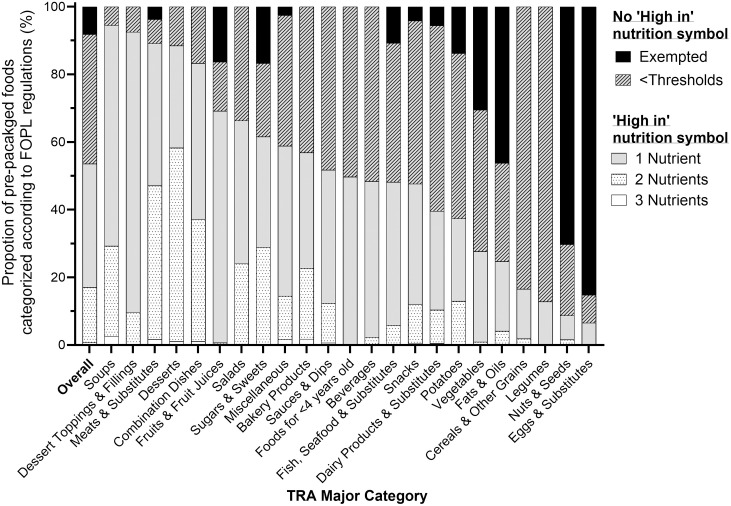
Proportion of pre-packaged foods categorized according to Canadian front-of-pack labelling (FOPL) regulations. Pre-packaged foods in Food Label Information and Price (FLIP) 2017 were used (n = 17,008). Proportions are presented overall and by Table of Reference Amounts for Food (TRA) major category [10]. FOPL regulations mandate pre-packaged foods to display a ‘High in’ nutrition symbol if they meet and/or exceed thresholds for nutrients-of-concern (saturated fat, total sugars, or sodium). Foods would not display a ‘High in’ nutrition symbol if they meet the exemption criteria (i.e., “Exempted”) or have nutrient levels below thresholds for all 3 nutrients-of-concern (i.e., “<Thresholds”). Foods would display a ‘High in’ nutrition symbol for meeting and/or exceeding thresholds for nutrient(s)-of-concern (i.e., 1-3 Nutrients). Abbreviations: FOPL, front-of-pack labelling; TRA, Table of Reference Amounts for Food.

Fig 1 and S1 Table show the proportion of foods classified according to FOPL regulations by TRA major and minor category, respectively. According to FOPL regulations, 53.7% of Canadian pre-packaged foods (n = 9,131) would display a ‘High in’ nutrition symbol for meeting and/or exceeding threshold levels for at least one nutrient-of-concern – 68.1% of these foods would display a symbol for one nutrient (n = 6,223/9,131), 30.5% for two nutrients (n = 2,786/9,131), and 1.3% for three nutrients (n = 122/9,131). Out of 46.3% of foods (n = 7,877) that would not display a ‘High in’ nutrition symbol, 17.4% (n = 1,370/7,877) would be exempted from FOPL regulations and 82.6% (n = 6,507/7,877) would have levels of nutrients-of-concern below thresholds. The top three categories with the highest proportion of foods that would display a ‘High in’ nutrition symbol were: Soups (94.5%, n = 449/475), Dessert Toppings & Fillings (92.5%, n = 87/94), and Meats & Substitutes (89.2%, n = 849/952). The top three categories with the highest proportion of foods that would not display a ‘High in’ nutrition symbol were: Eggs & Substitutes (93.4%, n = 57/61), Nuts & Seeds (91.3%, n = 230/252); and Legumes (87.2%, n = 163/187).

S2 Table shows the proportion of foods that would display each type of nutrient-of-concern for meeting and/or exceeding thresholds overall and by TRA major category. Overall, 20.9% of foods would display a ‘High in’ nutrition symbol for saturated fat (n = 3,563), 23.8% for total sugars (n = 4,055), and 26.7% for sodium (n = 4,543). The top three categories with the highest proportion of foods that would display a ‘High in’ nutrition symbol for saturated fat were: Desserts (56.3%, n = 382/679), Meats & Substitutes (51.3%; n = 488/952), and Sugars & Sweets (39.9%, n = 420/1,052). The top three categories with the highest proportion of foods that would display a ‘High in’ nutrition symbol for total sugars were: Dessert Toppings & Fillings (92.5%, n = 87/94), Desserts (81.1%; n = 551/679), and Fruits & Fruit Juices (68.1%, n = 712/1,045). The top three categories with the highest proportion of foods that would display a ‘High in’ nutrition symbol for sodium were: Soups (94.1%, n = 447/475), Meats & Substitutes (82.1%; n = 782/952), and Combination Dishes (77.7%, n = 824/1,061).

### Assessment of pre-packaged foods using the CFSS

**Fig 2:**
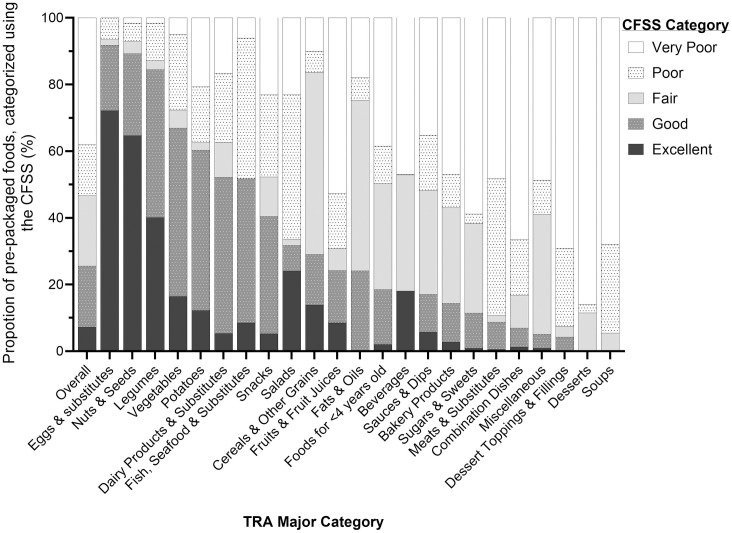
Proportion of pre-packaged foods categorized using the Canadian Food Scoring System (CFSS). Pre-packaged foods in Food Label Information and Price (FLIP) 2017 were used (n = 17,008). Proportions are presented overall and by Table of Reference Amounts for Food (TRA) major category [10]. The CFSS assessed the alignment of individual foods with the recommendations of Canada’s food guide (CFG) and Canada’s Dietary Guidelines for Health Professionals and Policymakers (CDG) using existing labelling regulations and standards [11]. The CFSS classified foods into one of five categories: ‘excellent,’ ‘good,’ ‘fair,’ ‘poor,’ or ‘very poor’ choice according to the recommendations of CFG and CDG. Abbreviations: CDG, Canada’s Dietary Guidelines for Health Professionals and Policymakers; CFG, Canada’s food guide; CFSS, Canadian Food Scoring System; FLIP, Food Label Information and Price; TRA, Table of Reference Amounts for Food.

Fig 2 and S3 Table show the proportion of foods classified according to the CFSS categories by TRA major and minor category, respectively. Overall, 7.2% of foods (n = 1,219) were classified as an ‘excellent’ choice, 18.3% (n = 3,110) as ‘good’, 21.2% (n = 3,600) as ‘fair,’ 15.3% (n = 2,598) as ‘poor,’ and 38.1% (n = 6,481) as ‘very poor.’ The top three categories with the highest proportion of foods classified as an ‘excellent’ or ‘good’ choice were: Eggs & Substitutes (91.8%, n = 56/61), Nuts & Seeds (89.3%, n = 225/252), and Legumes (84.5%, n = 158/187). The top three categories with the highest proportion of foods classified as a ‘poor’ or ‘very poor’ choice were: Soups (94.5%, n = 449/475), Dessert Toppings & Fillings (92.5%, n = 87/94), and Meats & Substitutes (89.2%, n = 849/952).

### Comparing FOPL regulations with the recommendations of CFG

S4 Table shows the mean nutritious food points (i.e., Step 1 of the CFSS) and the mean final CFSS scores (i.e., Step 1 + Step 2) by TRA minor category. The overall mean nutritious food point was 30.2 ± 34.2 (range: 0–100), indicating low amounts of nutritious foods recommended by CFG. In fact, 56.6% of foods (n = 9,632) had low amounts of nutritious foods (≤40 points), while 27.8% (n = 4,723) had high amounts of nutritious foods recommended by CFG (>60 points). Approximately 60% of the categories (n = 14/23) had mean nutritious food points ≤40 with the lowest mean points from: Desserts (1.4 ± 8.1), Soups (9.2 ± 17.1), and Miscellaneous (10.2 ± 22.0). The top three categories with the highest mean nutritious food points were: Nuts & Seeds (85.2 ± 19.1), Legumes (78.2 ± 21.4), and Eggs & Substitutes (75.1 ± 17.7).

The mean final CFSS score was 47.5 ± 25.6 (range: 10–100). Similar to the nutritious food points, the top three categories with the highest mean CFSS scores were Nuts & Seeds (88.9 ± 17.4), Eggs & Substitutes (85.1 ± 15.1), and Legumes (83.9 ± 19.1), and the top three categories with the lowest scores were: Desserts (23.8 ± 10.8), Soups (26.2 ± 8.7), Dessert Toppings & Fillings (31.8 ± 14.5).

S5 Table shows the mean nutritious food points (i.e., Step 1 of the CFSS) by the 5-level FOPL regulation categories overall and by TRA major category. Overall, foods that would be exempted from FOPL regulations had the highest mean nutritious food point of 61.0 ± 36.9 (range: 0–100), followed by foods that would not display a ‘High in’ nutrition symbol for having nutrient levels below thresholds (36.9 ± 35.6), and foods that would display a ‘High in’ nutrition symbol for 1–3 nutrients (23.4 ± 30.6, 18.8 ± 26.6, and 17.9 ± 28.4, respectively). The mean nutritious food points were significantly different by the 5-level FOPL categories overall and in all food categories (p ≤ 0.04) except for Cereals & Other Grains (p = 0.11), Miscellaneous (p = 0.06), and Soups (p = 0.45). The mean nutritious food points tended to be higher among foods that would not display a ‘High in’ nutrition symbol (i.e., exempted from FOPL regulations or having below threshold levels of nutrients-of-concern); however, it was not consistently seen in some food categories (e.g., Dairy Products & Substitutes, Desserts, Dessert Fillings & Toppings, Fats & Oils, Seafood & Substitutes, Nuts & Seeds).

S6 Table shows the mean final CFSS scores by the 5-level FOPL regulation categories, overall and by TRA major category. Foods that would be exempted from FOPL regulations had the highest mean final CFSS score of 80.5 ± 18.5 (range: 10–100), followed by foods that would not display a ‘High in’ nutrition symbol for having below thresholds (68.5 ± 17.8), and foods that would display a ‘High in’ nutrition symbol for 1–3 nutrients ( 30.9 ± 7.6, 20.8 ± 4.7, and 11.8 ± 2.8, respectively). Foods that would not display a ‘High in’ nutrition symbol (both exempted and <thresholds for nutrients-of-concern) had higher CFSS scores compared to those that would display a ‘High in’ nutrition symbol overall and across all food categories (p < 0.001).

**Fig 3:**
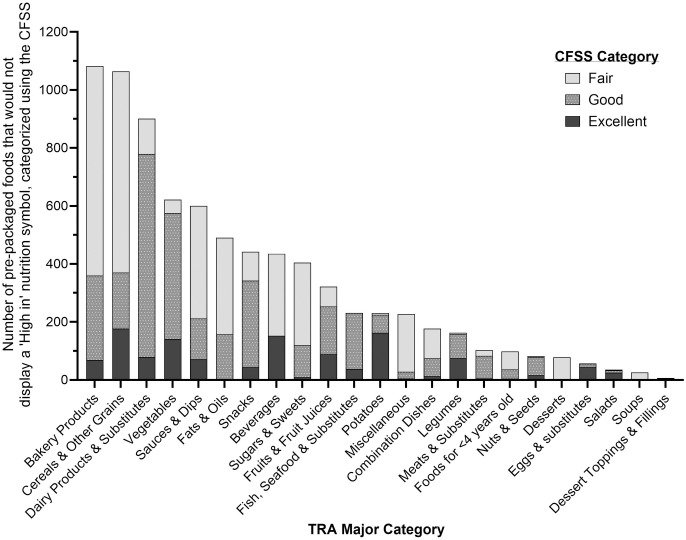
Number of pre-packaged foods that would not display a ‘High in’ nutrition symbol categorized using the Canadian Food Scoring System (CFSS). Pre-packaged foods in Food Label Information and Price (FLIP) 2017 that would not display a ‘High in’ nutrition symbol according to Canadian front-of-pack labelling regulations (i.e., foods exempted from the regulations and foods that have below thresholds for all three nutrients-of-concern) [10] were classified according to the CFSS categories [11]. n = 7,877. Proportions are presented by Table of Reference Amounts for Food (TRA) major category [250]. Sample sizes for each category are shown in brackets. The CFSS assessed the alignment of individual foods with the recommendations of Canada’s food guide (CFG) and Canada’s Dietary Guidelines for Health Professionals and Policymakers (CDG) using existing labelling regulations and standards [11]. The CFSS classified foods into one of five categories: ‘excellent,’ ‘good,’ ‘fair,’ ‘poor,’ or ‘very poor’ choice according to the recommendations of CFG and CDG. No foods that would not display a ‘High in’ nutrition symbol were rated as a ‘poor’ or ‘very poor’ choice. Abbreviations: CDG, Canada’s Dietary Guidelines for Health Professionals and Policymakers; CFG, Canada’s food guide; CFSS, Canadian Food Scoring System; FLIP, Food Label Information and Price; TRA, Table of Reference Amounts for Food.

Fig 3 shows the proportion of foods that would not display a ‘High in’ nutrition symbol categorized by the CFSS by TRA major category. Among foods that would not display a ‘High in’ nutrition symbol (n = 7,877), 15.5% (n = 1,219) were rated as an ‘excellent’ choice according to the CFSS, 39.5% as ‘good’ (n = 3,110), and 45.0% (n = 3,548) as ‘fair.’ None of the foods that would not display a ‘High in’ nutrition symbol were rated as a ‘poor’ or ‘very poor’ choice. More than half of foods that would not display a ‘High in’ nutrition symbol in Bakery Products, Beverages, Cereals & Other Grains, Desserts, Fats & Oils, Miscellaneous, Combination Dishes, Sauces & Dips, Soups, Sugars & Sweets, and Foods for <4-years-old food categories were identified as a ‘fair’ choice. The majority of foods (≥80%) that would not display a ‘High in’ nutrition symbol in Eggs & Substitutes, Nuts & Seeds, and Salads food categories were identified as an ‘excellent’ choice.

Among foods that would display a ‘High in’ nutrition symbol (n = 9,131), 0.6% (n = 52) were rated as a ‘fair’ choice according to the CFSS, 28.5% as ‘poor’ (n = 2,598), and 71.0% (n = 6,481) as ‘very poor.’

## Discussion

The objective of this study was to evaluate the potential impact of FOPL regulations on the Canadian pre-packaged food supply and the extent to which the regulations could support healthy food choices according to the recommendations of CFG. Approximately 54% of pre-packaged foods in Canada were ‘high in’ at least one nutrient-of-concern, which will be highlighted with a ‘High in’ nutrition symbol when FOPL regulations are implemented. However, only about a quarter of the foods were identified as a ‘good’ or ‘excellent’ choice according to the CFSS, due to the overall low amounts of nutritious foods recommended by CFG. As a result, nearly half of the foods that would not display a ‘High in’ nutrition symbol contained low amounts of nutritious foods recommended by CFG, highlighting potential gaps in FOPL regulations for identifying ‘healthier’ foods aligned well with the recommendations of CFG.

Similar to previous studies examining the quality of the Canadian pre-packaged food supply, our findings showed a large proportion of pre-packaged foods would display a ‘High in’ nutrition symbol according to Canadian FOPL regulations. FOPL has been shown to improve unhealthy diets by promoting manufacturer-driven reformulation [13] and encouraging ‘healthier’ purchasing behaviours by consumers [14–16]. As FOPL regulations are implemented in Canada, future studies examining the changes to the quality of the pre-packaged food supply will be needed to evaluate the effectiveness of the regulations. Particularly as our findings showed the final FOPL regulations were changed from the draft regulations pre-published in 2018 [6], resulting in fewer products in the Canadian food supply that would be required to display a ‘High in’ nutrition symbol (i.e., 64% according to the proposed regulations pre-published in 2018 [6] vs. 54% according to the final regulations published in 2022, as shown in the current analysis). Although FOPL regulations will help consumers easily and quickly identify products that are ‘high in’ nutrients-of-concern to help curtail their intakes, the effectiveness of the regulations must be examined to evaluate the regulations and strengthen them, as needed.

In addition to the high prevalence of pre-packaged foods ‘high in’ nutrients-of-concern, our findings also revealed a high prevalence of foods that did not align well with the nutritious food recommendations in CFG. In fact, many food categories had low mean nutritious food points (≤40 out of 100), highlighting the need to improve the quality of the pre-packaged food supply to support Canadians’ ability to easily make healthier food choices. Healthy dietary patterns encompass not only reducing intakes of nutrients-of-concern, but also include high intakes of nutritious foods, including whole grains and plant-based protein foods [7,12]. Pre-packaged foods, which typically undergo some level of processing, can offer convenient, available, and affordable ways for many Canadians to access perishable foods with health benefits (e.g., fruits, vegetables, nuts); however, many pre-packaged foods, partly related to processing, may be energy-dense with high levels of fats, sugars, and sodium. Since FOPL regulations solely target levels of nutrients-of-concern in pre-packaged foods, additional strategies will be needed to address other aspects of these foods and improve their overall quality in order for them to be part of healthy dietary patterns for Canadians. Further, the nutrient-specific approach of FOPL regulations may also lead to unintended consequences that do not effectively support the reformulation of ‘less healthy’ foods to ‘healthy’ foods. For instance, following the implementation of the Chilean Food Labelling and Advertising Law, the use of non-nutritive sweeteners increased as sugar levels decreased, suggesting that foods ‘high in’ sugars were reformulated with non-nutritive sweeteners to avoid displaying a front-of-pack warning label [17]. To better examine how FOPL regulations drive both intended outcomes and unintended consequences by decreasing the availability of nutrients-of-concern, nutrient levels and other components or additives to foods, including nutrients-to-encourage and other ingredients (e.g., non-nutritive sweeteners) must be monitored over time.

While foods that would not display a ‘High in’ nutrition symbol tended to have more nutritious foods recommended by CFG compared to those that would display a ‘High in’ nutrition symbol, the availability of nutritious foods recommended by CFG varied by food category. Our findings may be, in part, related to the exemption criteria for FOPL regulations (i.e., health-, technical-, and practical-related reasons), which may not necessarily be aligned with CFG recommendations. For instance, culinary ingredients that would meet the technical-related exemption criteria (e.g., table salt, honey, butter) are sources of nutrients-of-concern, accounting for 13–20% of intakes of saturated fat, sodium, and free sugars among Canadian adults [18]. Further, nutrients-of-concern levels associated with FOPL regulations are not necessarily associated with nutritious foods recommended by CFG (e.g., whole grains, plant-based protein). This is particularly concerning as between-category comparisons with clear features of CFG recommendations may be straightforward (e.g., Legumes, Fruits, Vegetables vs. Desserts, Sauces & Dips), within-category comparisons may be more challenging, especially for food categories containing a variety of different ingredients (e.g., Bakery Products, Cereals & Other Grains, Snacks). Similar to our findings, commonly-consumed breads sold in Quebec, Canada, had significant variations in grain composition and nutritional quality [19]. As FOPL regulations primarily rely on nutrient thresholds to identify ‘less healthy’ foods, determining the healthfulness of foods and their alignment with the recommendations of CFG (e.g., whole grains) within certain food categories may be challenging for many consumers. There is a need for a tool, such as the CFSS, that can complement FOPL regulations to easily identify individual foods’ alignment with the recommendations of CFG beyond those related to levels of nutrients-of-concern.

Conversely, some foods that would need to display a ‘High in’ nutrition symbol also had nutritious food components recommended by CFG (e.g., whole grains, plant-based protein), which may lead to consumer confusion in determining the healthfulness of foods as FOPL regulations are implemented. Unlike the Chilean Food Labelling and Advertising Law, which prohibits marketing of foods that would display FOPL symbol(s) to children [20] and has shown to decrease the purchases of nutrients-of-concern [21], Canadian FOPL regulations have no restrictions on marketing of foods that would display a ‘High in’ nutrition symbol. In other words, foods that would display a ‘High in’ nutrition symbol may be promoted to children, display Health Canada regulated voluntary claims (e.g., ‘source of fibre’), and use other marketing strategies (e.g., ‘plant-based’, ‘made with whole grains’, vignettes of fruits) on their packaging. Previous studies have shown that voluntary nutrition and health claims can create a ‘health halo’ effect, leading to misperceptions about the healthfulness of foods [22]. Other non-government-regulated labelling and marketing strategies that emphasize different aspects of foods, including food components (e.g., Certified Plant-Based program by Plant-Based Foods of Canada and Plant-Based Foods Association [23]; Walmart’s Ingredient Wise program [24]) and ‘eco-labels’ (e.g., FAIRTRADE Mark by the International Fairtrade System [25]), may influence consumers’ purchasing decisions beyond the levels of nutrients-of-concern. Health Canada’s ‘High in’ nutrition symbol may also compete with other factors that influence purchasing decisions, including price, brand loyalty, dietary restrictions, and health conditions [26]. Further research is warranted to explore potential barriers and facilitators of consumers’ purchasing decisions related to Health Canada’s ‘High in’ nutrition symbol, such as marketing strategies, price, and dietary restrictions, to strengthen the regulations accordingly.

The CFSS, a nutrient profile model that combines both nutritious foods recommended by CFG and thresholds for nutrients-of-concern, showed better discriminatory ability to determine the healthfulness of foods compared to FOPL regulations, which primarily focused on the levels of nutrients-of-concern. Of particular concern, about half of foods that would not display a ‘High in’ nutrition symbol contained low amounts of nutritious foods recommended by CFG. This demonstrates the potential challenge that FOPL regulations will have in further distinguishing the healthfulness of foods beyond the levels of nutrients-of-concern. Focusing solely on nutrient-based recommendations overlooks important nutritious foods associated with improved health outcomes. For example, although approximately 48% of foods in both the *Beverages* and *Seafood & Substitutes* categories met and/or exceeded thresholds for at least one nutrient-of-concern (Fig 1), the higher mean nutritious food point of the Seafood & Substitutes category compared to the Beverages category (53.6 ± 10.7 vs. 19.5 ± 39; S4 Table) resulted in a higher prevalence of foods identified as a ‘good’ or ‘excellent’ choice according to the CFSS in the *Seafood & Substitutes* category compared to the *Beverages* category (51.8% vs. 18.0%; Fig 2). Similarly, following solely food-based recommendations alone could overlook important nutrient considerations. For instance, despite similar mean nutritious food point for the *Beverages* and *Dessert Toppings & Fillings* categories (19.5 ± 39.0 vs. 19.5 ± 32.3; S4 Table), almost all foods in the *Dessert Toppings & Fillings* category (92.5%) met and/or exceeded thresholds for at least one nutrient-of-concern, while nearly half of the foods in the *Beverages* category (48.4%) met and/or exceeded thresholds (Fig 1). As a result, there was a greater prevalence of foods ranked as a ‘good’ or ‘excellent’ choice according to the CFSS in the *Beverages* category compared to the *Dessert Toppings & Fillings* category (18% vs. 4.3%, respectively; Fig 2). Furthermore, using the CFSS, foods in the *Ready-to-eat cereals, puffed and coated* category with whole grain ingredients and low-to-moderate levels of nutrients-of-concern (‘excellent’ or ‘good’ choice) could be differentiated from refined grain products with low-to-moderate levels of nutrients-of-concern (‘fair’ choice), and refined or whole grain products with high levels of nutrient(s)-of-concern (‘very poor’ to ‘poor’ choice). Therefore, the CFSS, which translated and integrated both food- and nutrient-based recommendations of the CFG, was better able to effectively discriminate the healthfulness of foods between and within food categories.

Although Canada and many other countries (e.g., Chile [20], Mexico [27], Israel [28]) use a binary FOPL system to identify ‘less healthy’ foods (i.e., ‘high in’ nutrients-of-concern), some countries are adopting other labelling regulations or a dual FOPL system to further differentiate ‘healthier’ foods. For instance, Israel uses mandatory ‘High in’ nutrition symbols to highlight foods high in nutrients-of-concern and a voluntary symbol to highlight foods that align with the Mediterranean diet [28]. The United States defines criteria for a voluntary ‘healthy’ nutrition claim, which is aligned with federal dietary guidelines (i.e., Dietary Guidelines for Americans) [29,30] and is currently developing a voluntary front-of-pack ‘healthy’ symbol [31]. Government-endorsed voluntary FOPL systems on food packages and/or other consumer-friendly tools (e.g., mobile apps [32]) that leverage a more comprehensive nutrient profile model such as the CFSS may further help Canadians to easily make healthy food choices and supplement the information that will be provided by FOPL regulations.

There are a few limitations to note. First, our analysis does not include fresh and other unpackaged foods that are significant parts of Canadians’ diets. Compared to the current analysis using the branded database of pre-packaged foods showing most of them to be identified as a ‘fair’ choice, previous analysis using the Canadian Nutrient File, a generic food composition database, that contains both fresh and pre-packaged foods showed a large proportion of foods without a ‘High in’ nutrition symbol to be an ‘excellent’ choice [11]. Considering the dietary guidance in CFG includes both pre-packaged and fresh foods [7], future analysis using a database of fresh and pre-packaged foods will provide a more comprehensive overview of how products in the food supply align with FOPL and the CFSS. Second, we did not assess the nutrient levels of foods. The Chilean Food Labelling and Advertising Law, which used a nutrient-specific approach similar to the Canadian FOPL regulations, has been shown to promote reductions in nutrients-of-concern in foods that fell just below regulatory thresholds [13]. Regular monitoring of nutrient levels in pre-packaged foods is warranted to evaluate the effectiveness of the regulations and strengthen them, as necessary. Third, we did not incorporate sales or consumption data related to the examined products. To monitor and evaluate the effectiveness of FOPL and CFG, purchasing and/or consumption data will be vital in understanding how these policies and regulations influence behaviours of Canadians. For instance, following the implementation of the Food Labelling and Advertising Law in Chile, household purchases of foods displaying ‘high in’ label(s) decreased [16,33]; such data and analyses in Canada will be needed to further evaluate the effectiveness of FOPL regulations.

## Conclusion

Our analyses showed that a high proportion of foods in the Canadian pre-packaged food supply would display a ‘High in’ nutrition symbol according to Canadian FOPL regulations for having ‘high’ levels of nutrient(s)-of-concern and had low amounts of nutritious foods recommended by CFG. Although FOPL regulations will help Canadians easily identify foods ‘high in’ nutrient(s)-of-concern when FOPL regulations are implemented, many foods that would not display a ‘High in’ nutrition symbol were not nutritious foods recommended by CFG. Additional public health strategies and tools that are accessible, affordable, and can complement FOPL are needed to further support healthy eating.

## Supporting information

S1 Table Number and proportion of pre-packaged foods classified according to front-of-pack labelling (FOPL) regulations. (PDF)

S2 Table Number and proportion of pre-packaged foods that would display a ‘High in’ nutrition symbol according to front-of-pack labelling (FOPL) regulations presented by the nutrient-of-concern type. (PDF)

S3 Table Number and proportion of pre-packaged foods categorized according to the Canadian Food Scoring System (CFSS). (PDF)

S4 Table Mean nutritious food points and final Canadian Food Scoring System (CFSS) scores of pre-packaged foods. (PDF)

S5 Table Mean nutritious food points of pre-packaged foods by front-of-pack labelling (FOPL) regulation category. (PDF)

S6 Table Mean Canadian Food Scoring System (CFSS) scores of pre-packaged foods by front-of-pack labelling (FOPL) regulation category. (PDF)
