# Supplementary material for: Assessing the extent to which front-of-pack labelling regulations could support healthy eating among Canadians
Source: PLoS One. 2025 Oct 8;20(10):e0330720. doi: 10.1371/journal.pone.0330720 (PMC12507316; doi:10.1371/journal.pone.0330720)
Supplement: S1 Table — (PDF) [file pone.0330720.s001.zip › Lee_CND FOPL_S3.pdf]

Assessing the extent to which front-of-pack labelling regulations could support healthy eating among Canadians
Lee JJ, Mulligan C, Jeong H, L'Abbe MR

S3 Table. Number and proportion of pre-packaged foods categorized according to the Canadian Food Scoring System.

TRA Category[*]	n	CFSS category, n (%) 'Very Poor'	'Poor'	'Fair'	'Good'	'Excellent'
**A. Bakery Products**						
A1. Bread, excluding sweet quick-type	289	103 (35.6%)	8 (2.8%)	127 (43.9%)	46 (15.9%)	5 (1.7%)
A2. Tea biscuits, scones, rolls, buns, etc.	235	73 (31.1%)	8 (3.4%)	118 (50.2%)	36 (15.3%)	0
A3. Bagels, naan, flat bread	80	51 (63.8%)	4 (5.0%)	19 (23.8%)	6 (7.5%)	0
A4. Brownies	29	17 (58.6%)	3 (10.3%)	6 (20.7%)	3 (10.3%)	0
A5. Heavy weight cake	64	64 (100.0%)	0	0	0	0
A6. Medium weight cake	101	97 (96.0%)	4 (4.0%)	0	0	0
A7. Light weight cake	5	5 (100.0%)	0	0	0	0
A8. Coffee cakes, doughnuts, sweet quick-type breads, etc.	82	64 (78.0%)	2 (2.4%)	15 (18.3%)	1 (1.2%)	0
A9. Muffins	34	33 (97.1%)	0	1 (2.9%)	0	0
A10. Cookies[†]	501	364 (72.7%)	47 (9.4%)	78 (15.6%)	10 (2.0%)	2 (0.4%)
A11. Accompaniment crackers	254	37 (14.6%)	7 (2.8%)	167 (65.7%)	30 (11.8%)	13 (5.1%)
A12. Snack crackers	66	35 (53.0%)	9 (13.6%)	14 (21.2%)	7 (10.6%)	1 (1.5%)
A13. Dry breads	87	26 (29.9%)	7 (8.0%)	31 (35.6%)	18 (20.7%)	5 (5.7%)
A14. Toaster pastries	11	6 (54.5%)	0	5 (45.5%)	0	0
A15. Ice cream cones	20	0	0	20 (100.0%)	0	0
A16. Croutons	37	2 (5.4%)	0	34 (91.9%)	1 (2.7%)	0
A17. French toast, pancakes, waffles	54	38 (70.4%)	8 (14.8%)	8 (14.8%)	0	0
A18. Grain-based bars with filling	105	21 (20.0%)	12 (11.4%)	27 (25.7%)	42 (40.0%)	3 (2.9%)
A19. Grain-based bars without filling	94	9 (9.6%)	23 (24.5%)	10 (10.6%)	30 (31.9%)	22 (23.4%)
A20. Energy and protein bars	205	29 (14.1%)	95 (46.3%)	18 (8.8%)	47 (22.9%)	16 (7.8%)
A21. Rice and corn cakes	35	7 (20.0%)	4 (11.4%)	8 (22.9%)	14 (40.0%)	2 (5.7%)
A22. Pies, pastries, etc.	79	70 (88.6%)	9 (11.4%)	0	0	0
A23. Pie crust	18	18 (100.0%)	0	0	0	0
A24. Pizza crust	13	3 (23.1%)	0	10 (76.9%)	0	0
A25. Taco shell	13	7 (53.8%)	0	6 (46.2%)	0	0
**Category A Total**	**2,511**	**1,179 (47.0%)**	**250 (10.0%)**	**722 (28.8%)**	**291 (11.6%)**	**69 (2.7%)**
**B. Beverages**						
B1. Carbonated and non-carbonated beverages[†]	713	371 (52.0%)	0	276 (38.7%)	0	66 (9.3%)
B3. Coffee[†,⁑,‡]	28	0	1 (3.6%)	5 (17.9%)	0	22 (78.6%)
B4. Tea	70	0	0	6 (8.6%)	0	64 (91.4%)
B5. Cocoa and hot chocolate beverages	32	25 (78.1%)	0	7 (21.9%)	0	0
**Category B Total**	**843**	**396 (47.0%)**	**1 (0.1%)**	**294 (34.9%)**	**0**	**152 (18.0%)**
**C. Cereals & Other Grains**						
C1. Hot breakfast cereals	118	1 (0.8%)	5 (4.2%)	9 (7.6%)	60 (50.8%)	43 (36.4%)
C2. Ready-to-eat cereals, puffed and uncoated	4	0	0	2 (50.0%)	2 (50.0%)	0
C3. Ready-to-eat cereals, puffed and coated without fruit or nuts	88	17 (19.3%)	13 (14.8%)	23 (26.1%)	26 (29.5%)	9 (10.2%)
C4. Ready-to-eat cereals, fruit and nut, granola type	169	6 (3.6%)	33 (19.5%)	22 (13.0%)	66 (39.1%)	42 (24.9%)
C5. Bran and wheat germ, milled flax, etc.	38	2 (5.3%)	0	26 (68.4%)	3 (7.9%)	7 (18.4%)
C6. Flours and cornmeal	66	1 (1.5%)	1 (1.5%)	55 (83.3%)	2 (3.0%)	7 (10.6%)
C7. Grains	279	59 (21.1%)	24 (8.6%)	129 (46.2%)	6 (2.2%)	61 (21.9%)
C8. Pastas	492	32 (6.5%)	4 (0.8%)	421 (85.6%)	28 (5.7%)	7 (1.4%)

TRA Category[*]	n	CFSS category, n (%) 'Very Poor'	'Poor'	'Fair'	'Good'	'Excellent'
C10. Starch	10	1 (9.1%)	0	10 (90.9%)	0	0
C11. Stuffing	118	9 (90.0%)	0	0	0	1 (10.0%)
**Category C Total**	**1,275**	**128 (10.0%)**	**80 (6.3%)**	**697 (54.7%)**	**193 (15.1%)**	**177 (13.9%)**
**D. Dairy Products & Substitutes**						
D1. Cheese	568	53 (9.3%)	75 (13.2%)	13 (2.3%)	427 (75.2%)	0
D2. Cottage cheese	21	0	2 (9.5%)	0	19 (90.5%)	0
D3. Cheese used as ingredient	14	0	7 (50.0%)	0	7 (50.0%)	0
D4. Hard cheese	44	5 (11.4%)	10 (22.7%)	1 (2.3%)	28 (63.6%)	0
D5. Quark	110	36 (32.7%)	9 (8.2%)	1 (0.9%)	64 (58.2%)	0
D6. Cream and cream substitutes	41	0	0	0	39 (95.1%)	2 (4.9%)
D7. Powder cream and cream substitutes	9	2 (22.2%)	0	7 (77.8%)	0	0
D8. Aerosol/whipped cream and cream substitutes	22	10 (45.5%)	0	12 (54.5%)	0	0
D10. Evaporated/condensed milk	17	0	7 (41.2%)	1 (5.9%)	4 (23.5%)	5 (29.4%)
D11. Milk, buttermilk, milk-based drinks, plant-based milk substitutes	200	28 (14.0%)	2 (1.0%)	96 (48.0%)	7 (3.5%)	67 (33.5%)
D12. Fermented dairy drinks	59	48 (81.4%)	0	11 (18.6%)	0	0
D13. Shakes and smoothies	25	2 (8.0%)	6 (24.0%)	12 (48.0%)	0	5 (20.0%)
D14. Sour cream	24	7 (29.2%)	6 (25.0%)	2 (8.3%)	9 (37.5%)	0
D15. Yogurt	338	58 (17.2%)	184 (54.4%)	0	96 (28.4%)	0
**Category D Total**	**1,492**	**249 (16.7%)**	**308 (20.6%)**	**156 (10.5%)**	**700 (46.9%)**	**79 (5.3%)**
**E. Desserts**						
E1. Ice cream, frozen yogurt, sherbet, etc. in tubs	277	276 (99.6%)	1 (0.4%)	0	0	0
E2 Ice cream, frozen yogurt, sherbet, etc. as cakes, cones	61	57 (93.4%)	0	4 (6.6%)	0	0
E3 Ice cream, frozen yogurt, sherbet, etc. as pops, bars	153	104 (68.0%)	3 (2.0%)	46 (30.1%)	0	0
E4. Sundaes	8	8 (100.0%)	0	0	0	0
E5. Custard, gelatin, pudding	180	139 (77.2%)	13 (7.2%)	27 (15.0%)	1 (0.6%)	0
**Category E Total**	**679**	**584 (86.0%)**	**17 (2.5%)**	**77 (11.3%)**	**1 (0.1%)**	**0**
**F. Dessert Toppings & Fillings**						
F1. Dessert toppings	29	26 (89.7%)	0	3 (10.3%)	0	0
F2. Cake frostings[‡]	35	35 (100.0%)	0	0	0	0
F3. Pie fillings	30	4 (13.3%)	22 (73.3%)	0	4 (13.3%)	0
**Category F Total**	**94**	**65 (69.1%)**	**22 (23.4%)**	**3 (3.2%)**	**4 (4.3%)**	**0**
**G. Eggs & Substitutes**						
G1. Egg mixtures	5	0	2 (40.0%)	0	3 (60.0%)	0
G2. Eggs	56	0	2 (3.6%)	1 (1.8%)	9 (16.1%)	44 (78.6%)
**Category G Total**	**61**	**0**	**4 (6.6%)**	**1 (1.6%)**	**12 (19.7%)**	**44 (72.1%)**
**H. Fats & Oils**						
H1. Butter, margarine, lard, etc.	112	0	0	110 (98.2%)	2 (1.8%)	0
H2. Vegetable oil	166	0	0	34 (20.5%)	132 (79.5%)	0
H4. Dressings for salad	283	117 (41.3%)	44 (15.5%)	113 (39.9%)	9 (3.2%)	0
H5. Mayonnaise and mayonnaise-type dressing	68	0	0	68 (100.0%)	0	0
H6. Spray oil	23	0	0	9 (39.1%)	14 (60.9%)	0
**Category H Total**	**652**	**117 (17.9%)**	**44 (6.7%)**	**334 (51.2%)**	**157 (24.1%)**	**0**
**I. Seafood & Substitutes**						

TRA Category*	n	CFSS category, n (%) 'Very Poor'	'Poor'	'Fair'	'Good'	'Excellent'
I1. Anchovies, caviar	9	1 (11.1%)	5 (55.6%)	0	3 (33.3%)	0
I2. Marine and freshwater animals with sauce	50	11 (22.0%)	31 (62.0%)	0	8 (16.0%)	0
I3. Marine and freshwater animals without sauce	191	15 (7.9%)	92 (48.2%)	0	52 (27.2%)	32 (16.8%)
I4. Canned marine and freshwater animals	147	0	27 (18.4%)	0	114 (77.6%)	6 (4.1%)
I5. Smoked/pickled marine and freshwater animals	49	0	33 (67.3%)	0	16 (32.7%)	0
**Category I Total**	**446**	**27 (6.1%)**	**188 (42.2%)**	**0**	**193 (43.3%)**	**38 (8.5%)**
**J. Fruits & Fruit Juices**						
J1. Fruits (fresh, frozen, canned, coated, and uncoated)	186	0	88 (47.3%)	4 (2.2%)	63 (33.9%)	31 (16.7%)
J2. Berries	15	0	0	0	3 (20.0%)	12 (80.0%)
J3. Melons	5	0	0	0	0	5 (100.0%)
J4. Avocados	1	0	0	0	1 (100.0%)	0
J5. Cranberries, lemons, limes	3	0	0	0	0	3 (100.0%)
J6. Fruit sauces and purees	65	0	19 (29.2%)	0	43 (66.2%)	3 (4.6%)
J7. Dried fruits	131	3 (2.3%)	44 (33.6%)	0	49 (37.4%)	35 (26.7%)
J8. Candied/pickled fruits	21	0	21 (100.0%)	0	0	0
J9. Fruits for garnish	5	0	0	0	5 (100.0%)	0
J11. Juices, nectars, fruit drinks	603	548 (90.9%)	0	55 (9.1%)	0	0
J12. Fruit juices used as ingredients	10	0	0	10 (100.0%)	0	0
**Category J Total**	**1,045**	**551 (52.7%)**	**172 (16.5%)**	**69 (6.6%)**	**164 (15.7%)**	**89 (8.5%)**
**K. Legumes**						
K1. Tofu or tempeh	23	0	6 (26.1%)	0	17 (73.9%)	0
K2. Beans, lentils, etc.	164	3 (1.8%)	15 (9.1%)	5 (3.0%)	66 (40.2%)	75 (45.7%)
**Category K Total**	**187**	**3 (1.6%)**	**21 (11.2%)**	**5 (2.7%)**	**83 (44.4%)**	**75 (40.1%)**
**L. Meats & Substitutes**						
L1. Pork rinds and bacon	41	19 (46.3%)	19 (46.3%)	0	3 (7.3%)	0
L2. Beef, pork and poultry breakfast strips	6	2 (33.3%)	2 (33.3%)	1 (16.7%)	1 (16.7%)	0
L3. Dried meat and poultry	96	81 (84.4%)	15 (15.6%)	0	0	0
L4. Luncheon meats	85	23 (27.1%)	57 (67.1%)	1 (1.2%)	4 (4.7%)	0
L5. Sausage products	160	117 (73.1%)	37 (23.1%)	3 (1.9%)	3 (1.9%)	0
L6. Cust of meat & poultry without sauce	125	35 (28.0%)	68 (54.4%)	1 (0.8%)	19 (15.2%)	2 (1.6%)
L7. Patties, ground meat with and without breading	214	72 (33.6%)	86 (40.2%)	14 (6.5%)	39 (18.2%)	3 (1.4%)
L8. Cured meats	86	16 (18.6%)	67 (77.9%)	0	3 (3.5%)	0
L9. Canned meats	27	14 (51.9%)	8 (29.6%)	0	5 (18.5%)	0
L10. Meat and poultry with sauce	112	80 (71.4%)	31 (27.7%)	0	1 (0.9%)	0
**Category L Total**	**952**	**459 (48.2%)**	**390 (41.0%)**	**20 (2.1%)**	**78 (8.2%)**	**5 (0.5%)**
**M. Miscellaneous**						
M1. Baking powder, baking soda, yeast†	25	3 (12.0%)	0	21 (84.0%)	1 (4.0%)	0
M2. Baking decoration	20	0	0	20 (100.0%)	0	0
M3. Bread crumbs	241	195 (80.9%)	14 (5.8%)	26 (10.8%)	6 (2.5%)	0
M5. Cocoa powder	5	0	0	5 (100.0%)	0	0
M7. Chewing gum	23	0	0	3 (100.0%)	0	0
M8. Salad and potato toppers	166	0	2 (8.7%)	11 (47.8%)	8 (34.8%)	2 (8.7%)
M9. Salt, salt substitutes†	36	49 (29.5%)	29 (17.5%)	84 (50.6%)	2 (1.2%)	2 (1.2%)
M10. Spices and herbs without salt	19	0	0	29 (80.6%)	6 (16.7%)	1 (2.8%)
M11. Coconut milk	14	19 (100.0%)	0	0	0	0

TRA Category*	n	CFSS category, n (%) ‘Very Poor’	‘Poor’	‘Fair’	‘Good’	‘Excellent’
M12. Dried coconut	25	3 (21.4%)	11 (78.6%)	0	0	0
**Category M Total**	**552**	**269 (48.7%)**	**56 (10.1%)**	**199 (36.1%)**	**23 (4.2%)**	**5 (0.9%)**
**N. Combination Dishes**						
N1. Combination dishes	529	328 (62.0%)	88 (16.6%)	61 (11.5%)	42 (7.9%)	10 (1.9%)
N2. Burritos, pizzas, sandwiches, meat pie, etc. †	408	306 (75.0%)	50 (12.3%)	34 (8.3%)	16 (3.9%)	2 (0.5%)
N3. Hors d'oeuvres	124	72 (58.1%)	40 (32.3%)	8 (6.5%)	3 (2.4%)	1 (0.8%)
**Category N Total**	**1,061**	**706 (66.5%)**	**178 (16.8%)**	**103 (9.7%)**	**61 (5.7%)**	**13 (1.2%)**
**O. Nuts & Seeds**						
O1. Nuts and seeds (not used for snacks)	140	0	0	3 (2.1%)	7 (5.0%)	130 (92.9%)
O2. Nut pastes and creams	7	3 (42.9%)	4 (57.1%)	0	0	0
O3. Nut butters	101	1 (1.0%)	10 (9.9%)	4 (4.0%)	55 (54.5%)	31 (30.7%)
O4. Nut flours	4	0	0	2 (50.0%)	0	2 (50.0%)
**Category O Total**	**252**	**4 (1.6%)**	**14 (5.6%)**	**9 (3.6%)**	**62 (24.6%)**	**163 (64.7%)**
**P. Potatoes**						
P1. French fries	65	0	13 (20.0%)	0	52 (80.0%)	0
P2. Mashed, stuffed, candied potatoes	37	27 (73.0%)	4 (10.8%)	3 (8.1%)	3 (8.1%)	0
P3. Fresh, canned, frozen potatoes	29	0	5 (17.2%)	0	8 (27.6%)	16 (55.2%)
**Category P Total**	**131**	**27 (20.6%)**	**22 (16.8%)**	**3 (2.3%)**	**63 (48.1%)**	**16 (12.2%)**
**Q. Salads**						
Q1. Salads	84	15 (17.9%)	40 (47.6%)	1 (1.2%)	6 (7.1%)	22 (26.2%)
Q3. Pasta, potato or grain-based salad	20	9 (45.0%)	5 (25.0%)	1 (5.0%)	2 (10.0%)	3 (15.0%)
**Category Q Total**	**104**	**24 (23.1%)**	**45 (43.3%)**	**2 (1.9%)**	**8 (7.7%)**	**25 (24.0%)**
**R. Sauces & Dips**						
R1. Dipping sauces	158	110 (69.6%)	9 (5.7%)	37 (23.4%)	2 (1.3%)	0
R2. Dips and spreads	183	32 (17.5%)	24 (13.1%)	22 (12.0%)	58 (31.7%)	47 (25.7%)
R3. Major main entrée sauce	225	100 (44.4%)	76 (33.8%)	16 (7.1%)	22 (9.8%)	11 (4.9%)
R4. Minor main entrée sauce	264	122 (46.2%)	37 (14.0%)	55 (20.8%)	41 (15.5%)	9 (3.4%)
R5. Major condiments†,‡	293	73 (24.9%)	60 (20.5%)	148 (50.5%)	11 (3.8%)	1 (0.3%)
R6. Minor condiments†,‡	121	1 (0.8%)	0	110 (90.9%)	7 (5.8%)	3 (2.5%)
**Category R Total**	**1,244**	**438 (35.2%)**	**206 (16.6%)**	**388 (31.2%)**	**141 (11.3%)**	**71 (5.7%)**
**S. Snacks**						
S1. Chips, pretzels, etc.†	562	150 (26.7%)	139 (24.7%)	94 (16.7%)	172 (30.6%)	7 (1.2%)
S2. Nuts or seeds (used as snacks)	252	19 (7.5%)	67 (26.6%)	6 (2.4%)	123 (48.8%)	37 (14.7%)
S3. Meat or poultry sticks	31	26 (83.9%)	2 (6.5%)	0	3 (9.7%)	0
**Category S Total**	**845**	**195 (23.1%)**	**208 (24.6%)**	**100 (11.8%)**	**298 (35.3%)**	**44 (5.2%)**
**T. Soups**						
T1. All varieties of soups (includes broth)	475	323 (68.0%)	126 (26.5%)	26 (5.5%)	0	0
**Category T Total**	**475**	**323 (68.0%)**	**126 (26.5%)**	**26 (5.5%)**	**0**	**0**
**U. Sugars & Sweets**						
U1. Candies, confectionaries, chocolates	534	515 (96.4%)	12 (2.2%)	7 (1.3%)	0	0
U3. Hard candies	20	7 (35.0%)	0	13 (65.0%)	0	0
U4. Baking candies	47	45 (95.7%)	2 (4.3%)	0	0	0
U5. Breath mints	2	0	0	2 (100.0%)	0	0
U7. Confectioner's or icing sugar	3	0	0	3 (100.0%)	0	0
U8. Honey, molasses, bread spreads	72	9 (12.5%)	1 (1.4%)	62 (86.1%)	0	0

		CFSS category, n (%)				
**TRA Category***	**n**	**'Very Poor'**	**'Poor'**	**'Fair'**	**'Good'**	**'Excellent'**
U9. Jams, jellies, fruit spreads	225	10 (4.4%)	13 (5.8%)	86 (38.2%)	107 (47.6%)	9 (4.0%)
U10. Fruit leather	20	19 (95.0%)	1 (5.0%)	0	0	0
U11. Marshmallows	12	12 (100.0%)	0	0	0	0
U12. Sugars†,‡	45	0	0	45 (100.0%)	0	0
U14. Syrups used as toppings	52	0	0	49 (94.2%)	3 (5.8%)	0
U15. Syrups used as ingredients	20	2 (10.0%)	0	17 (85.0%)	1 (5.0%)	0
**Category U Total**	**1,052**	**619 (58.8%)**	**29 (2.8%)**	**284 (27.0%)**	**111 (10.5%)**	**9 (0.9%)**
**V. Vegetables**						
V1. Vegetables without sauce	407	0	25 (6.1%)	0	263 (64.6%)	119 (29.2%)
V2. Vegetables with sauce	10	4 (40.0%)	3 (30.0%)	0	1 (10.0%)	2 (20.0%)
V3. Vegetables used for garnishing/flavouring†,‡	27	0	0	0	26 (96.3%)	1 (3.7%)
V4. Chili pepper & green onion†	42	2 (4.8%)	22 (52.4%)	0	15 (35.7%)	3 (7.1%)
V5. Seaweed, dehydrated mushrooms	19	0	0	0	8 (42.1%)	11 (57.9%)
V6. Sprouts	1	0	0	0	0	1 (100.0%)
V7. Vegetable juice and drink	58	27 (46.6%)	0	31 (53.4%)	0	0
V8. Olives	65	2 (3.1%)	38 (58.5%)	0	25 (38.5%)	0
V9. Sun-dried tomatoes and other pickled or oil-packed vegetables	170	3 (1.8%)	106 (62.4%)	0	59 (34.7%)	2 (1.2%)
V10. Relish	15	0	1 (6.7%)	1 (6.7%)	11 (73.3%)	2 (13.3%)
V11. Vegetable paste	12	0	0	2 (16.7%)	10 (83.3%)	0
V12. Vegetable sauce or purée	34	5 (14.7%)	0	12 (35.3%)	17 (50.0%)	0
**Category V Total**	**860**	**43 (5.0%)**	**195 (22.7%)**	**46 (5.3%)**	**435 (50.6%)**	**141 (16.4%)**
**W. Foods for <4 years old⁂**						
W1. Cereals to be prepared†	41	11 (26.8%)	9 (22.0%)	4 (9.8%)	13 (31.7%)	4 (9.8%)
W2. Ready-to-eat cereals and cereal bars†	9	0	9 (100.0%)	0	0	0
W3. Cookies, biscuits, etc. †	46	3 (6.5%)	3 (6.5%)	22 (47.8%)	18 (39.1%)	0
W4. Strained meat, desserts, combination dishes†	89	59 (66.3%)	1 (1.1%)	28 (31.5%)	1 (1.1%)	0
W5. Combination dishes†	8	0	0	8 (100.0%)	0	0
W6. Juices†	2	2 (100.0%)	0	0	0	0
**Category W Total**	**195**	**75 (38.5%)**	**22 (11.3%)**	**62 (31.8%)**	**32 (16.4%)**	**4 (2.1%)**
**OVERALL TOTAL**	**17,008**	**6,481 (38.1%)**	**2,598 (15.3%)**	**3,600 (21.2%)**	**3,110 (18.3%)**	**1,219 (7.2%)**

All values are presented as n (%). Pre-packaged foods in Food Label Information and Price 2017 were used in the analyses (n=17,008). The CFSS assessed the alignment of individual foods with the recommendations of Canada's food guide (CFG) and Canada's Dietary Guidelines for Health Professionals and Policymakers (CDG) using existing labelling regulations and standards [1]. The CFSS classified foods into one of five categories: 'excellent,' 'good,' 'fair,' 'poor,' or 'very poor' choice according to the recommendations of CFG and CDG. *Health Canada's Table of Reference Amounts for Food [2] was used to define food categories. †Indicates categories with products that were missing values for saturated fat (n=217; 1.3% overall). ‡Indicates categories with products that were missing values for sugars (n=5; 0.03% overall). ‡Indicates categories with products that were missing values for sodium (n=10; 0.06% overall). ⁂Although foods for <1-year-olds would be exempted from front-of-pack labelling regulations [3], all foods for <4-year-olds with a Nutrition Facts table were included as only the minimum age for consumption (e.g., ≥6-month-olds), not maximum age for consumption, are indicated in these foods. Abbreviations: CDG, Canada's Dietary Guidelines for Health Professionals and Policymakers; CFG, Canada's food guide; CFSS, Canadian Food Scoring System; TRA, Table of Reference Amounts for Food.

Assessing the extent to which front-of-pack labelling regulations could support healthy eating among Canadians
Lee JJ, Mulligan C, Jeong H, L'Abbe MR

References:

1. Lee JJ, Mulligan C, L'Abbé MR. Development and validity testing of the Canadian Food Scoring System (CFSS), a nutrient profile model based on the recommendations of Canada's food guide 2019. Appl Physiol Nutr Metab. 2024. doi: 10.1139/apnm-2024-0034 %M 39013203.
2. Health Canada. Table of Reference Amounts for Food. 2016 [cited 2019 July 15]. Available from: https://www.canada.ca/en/health-canada/services/technical-documents-labelling-requirements/table-reference-amount-food-2016.html.
3. Government of Canada. Regulations Amending the Food and Drug Regulations (Nutrition Symbols, Other Labelling Provisions, Vitamin D and Hydrogenated Fats or Oils): SOR/2022-168. Ottawa: Government of Canada; 2022 [cited 2022 July 30]. Available from: https://canadagazette.gc.ca/rp-pr/p2/2022/2022-07-20/html/sor-dors168-eng.html.
