# Supplementary material for: Assessing the extent to which front-of-pack labelling regulations could support healthy eating among Canadians
Source: PLoS One. 2025 Oct 8;20(10):e0330720. doi: 10.1371/journal.pone.0330720 (PMC12507316; doi:10.1371/journal.pone.0330720)
Supplement: S1 Table — (PDF) [file pone.0330720.s001.zip › Lee_CND FOPL_S4.pdf]

S4 Table. Mean nutritious food points and final Canadian Food Scoring System scores of pre-packaged foods.

TRA Category*	n	Nutritious food point (i.e., Step 1), means±SD	Final CFSS score, means±SD
**A. Bakery Products**			
A1. Bread, excluding sweet quick-type	289	14.6±28.6	47.2±21.4
A2. Tea biscuits, scones, rolls, buns, etc.	235	12.8±26.4	47.1±19.8
A3. Bagels, naan, flat bread	80	8.1±22.3	36.1±17.7
A4. Brownies	29	12.8±26.8	35.9±20.8
A5. Heavy weight cake	64	4.4±16.1	16.1±4.5
A6. Medium weight cake	101	4.9±15.8	19.6±4.9
A7. Light weight cake	5	0±0	23.5±3.4
A8. Coffee cakes, doughnuts, sweet quick-type breads, etc.	82	3±13.5	28.6±12.9
A9. Muffins	34	0±0	17.4±7.1
A10. Cookies†	501	8.9±22	28.7±15.1
A11. Accompaniment crackers	254	16.7±29.4	53.6±18.6
A12. Snack crackers	66	19.1±31.8	39.9±20.8
A13. Dry breads	87	24.4±33.4	50.9±23.8
A14. Toaster pastries	11	0±0	36.4±13.1
A15. Ice cream cones	20	0±0	50.0±0
A16. Croutons	37	1.9±11.5	49.6±8.3
A17. French toast, pancakes, waffles	54	3.0±7.2	29.0±9.3
A18. Grain-based bars with filling	105	39.9±34.6	59.2±25.7
A19. Grain-based bars without filling	94	60.9±31.8	66.9±26.4
A20. Energy and protein bars	205	58.4±27.5	53.5±23.2
A21. Rice and corn cakes	35	41.1±36.4	60.7±25.3
A22. Pies, pastries, etc.	79	29.2±34.6	22.9±9
A23. Pie crust	18	0±0	25.0±0
A24. Pizza crust	13	0±0	44.2±11.0
A25. Taco shell	13	0±0	35.4±14.3
**Category A Total**	**2,511**	**18.8±31**	**41.2±22.7**
**B. Beverages**			
B1. Carbonated and non-carbonated beverages†	713	9.3±29.0	41.5±22.2
B3. Coffee†,⁑,‡	28	100.0±0	88.8±22.1
B4. Tea	70	100.0±0	95.7±14.1
B5. Cocoa and hot chocolate beverages	32	0±0	29.5±11.3
**Category B Total**	**843**	**19.5±39**	**47.1±27.3**
**C. Cereals & Other Grains**			
C1. Hot breakfast cereals	118	73.8±25.9	84.9±16.3
C2. Ready-to-eat cereals, puffed and uncoated	4	40.0±35.6	70.0±17.8
C3. Ready-to-eat cereals, puffed and coated without fruit or nuts	88	40.8±36.4	59.2±25.2
C4. Ready-to-eat cereals, fruit and nut, granola type	169	65.9±27.4	73.1±22.2
C5. Bran and wheat germ, milled flax, etc.	38	33.9±44.3	61.7±21.8
C6. Flours and cornmeal	66	15.0±32.4	56.5±16.4
C7. Grains	279	26.6±40.7	55.2±26.4
C8. Pastas	492	7.9±20.5	51.6±12.6
C10. Starch	10	1.8±6.0	48.6±8.4
C11. Stuffing	118	9.0±28.5	32.0±22.1
**Category C Total**	**1,275**	**29.2±38.3**	**59.3±22**
**D. Dairy Products & Substitutes**			
D1. Cheese	568	47.5±11.6	64.8±18.3
D2. Cottage cheese	21	50.0±0	71.4±11.3
D3. Cheese used as ingredient	14	50.0±0	56.3±19.5
D4. Hard cheese	44	49.4±8.5	60.2±20.2
D5. Quark	110	50.4±8.5	56.0±22.8

TRA Category*	n	Nutritious food point (i.e., Step 1), means±SD	Final CFSS score, means±SD
D6. Cream and cream substitutes	41	76.2±5.5	88.1±2.7
D7. Powder cream and cream substitutes	9	0±0	44.4±11.0
D8. Aerosol/whipped cream and cream substitutes	22	0±0	38.6±12.7
D10. Evaporated/condensed milk	17	77.9±23.2	71.0±26.1
D11. Milk, buttermilk, milk-based drinks, plant-based milk substitutes	200	51.3±48.8	64.3±28.1
D12. Fermented dairy drinks	59	0±0	29.5±9.9
D13. Shakes and smoothies	25	88.0±33.2	54.2±24.9
D14. Sour cream	24	31.3±24.7	49±21.8
D15. Yogurt	338	50.1±10.6	46.8±19.8
**Category D Total**	**1,492**	**47.6±25.3**	**58.2±23**
**E. Desserts**			
E1. Ice cream, frozen yogurt, sherbet, etc. in tubs	277	0.2±2.1	19.0±3.2
E2 Ice cream, frozen yogurt, sherbet, etc. as cakes, cones	61	0.7±3.6	21.3±9
E3 Ice cream, frozen yogurt, sherbet, etc. as pops, bars	153	1.2±8.3	30.0±13.9
E4. Sundaes	8	0±0	17.5±0
E5. Custard, gelatin, pudding	180	3.6±13.3	27.0±12.4
**Category E Total**	**679**	**1.4±8.2**	**23.8±10.8**
**F. Dessert Toppings & Fillings**			
F1. Dessert toppings	29	0±0	26.6±8.5
F2. Cake frostings‡	35	0±0	24.1±2.4
F3. Pie fillings	30	61.0±26.8	45.7±17.3
**Category F Total**	**94**	**19.5±32.3**	**31.8±14.5**
**G. Eggs & Substitutes**			
G1. Egg mixtures	5	58.0±11.0	64±24.5
G2. Eggs	56	76.6±17.5	87±12.7
**Category G Total**	**61**	**75.1±17.7**	**85.1±15.1**
**H. Fats & Oils**			
H1. Butter, margarine, lard, etc.	112	6.7±13.2	53.3±6.6
H2. Vegetable oil	166	45.2±10.5	72.6±5.2
H4. Dressings for salad	283	13.0±16.6	39.7±16.6
H5. Mayonnaise and mayonnaise-type dressing	68	17.2±14.9	58.6±7.5
H6. Spray oil	23	41.3±11.4	70.7±5.7
**Category H Total**	**652**	**21.5±20.8**	**53.5±18.1**
**I. Seafood & Substitutes**			
I1. Anchovies, caviar	9	44.4±16.7	48.6±20.2
I2. Marine and freshwater animals with sauce	50	51.9±6.8	41.2±15.9
I3. Marine and freshwater animals without sauce	191	56.1±13.2	56.1±23.6
I4. Canned marine and freshwater animals	147	51.8±7.6	68.9±15.1
I5. Smoked/pickled marine and freshwater animals	49	52.4±6.6	50.4±17.4
**Category I Total**	**446**	**53.6±10.7**	**57.9±21.5**
**J. Fruits & Fruit Juices**			
J1. Fruits (fresh, frozen, canned, coated, and uncoated)	186	72.4±17.5	66.4±24.2
J2. Berries	15	94±12.4	97.0±6.2
J3. Melons	5	92±4.5	96.0±2.2
J4. Avocados	1	70±12.6	85.0±0
J5. Cranberries, lemons, limes	3	100±0	100.0±0
J6. Fruit sauces and purees	65	71.2±4.8	73.1±19.8
J7. Dried fruits	131	77.6±12.6	73.2±24.4
J8. Candied/pickled fruits	21	71.9±6	43.0±1.5

TRA Category*	n	Nutritious food point (i.e., Step 1), means±SD	Final CFSS score, means±SD
J9. Fruits for garnish	5	70±0	85.0±0
J11. Juices, nectars, fruit drinks	603	0±0	27.2±7.2
J12. Fruit juices used as ingredients	10	0±0	50.0±0
**Category J Total**	**1,045**	**31.0±38.1**	**45.2±26.7**
**K. Legumes**			
K1. Tofu or tempeh	23	70±0	73.9±19.1
K2. Beans, lentils, etc.	164	79.3±22.6	85.3±18.8
**Category K Total**	**187**	**78.2±21.4**	**83.9±19.1**
**L. Meats & Substitutes**			
L1. Pork rinds and bacon	41	50.5±3.1	35.2±12.7
L2. Beef, pork and poultry breakfast strips	6	36.7±21.6	43.5±19.9
L3. Dried meat and poultry	96	49.7±5.5	28±4.1
L4. Luncheon meats	85	49.1±6.3	36.3±10.1
L5. Sausage products	160	47.9±8.4	30.1±8.8
L6. Cust of meat & poultry without sauce	125	51.7±13.8	42.3±19.6
L7. Patties, ground meat with and without breading	214	52.1±16.8	44.5±21.7
L8. Cured meats	86	49.4±5.4	36.7±8.6
L9. Canned meats	27	48.1±9.6	38.3±18.7
L10. Meat and poultry with sauce	112	45.8±13.9	27.6±8.8
**Category L Total**	**952**	**49.6±11.9**	**36.1±16**
**M. Miscellaneous**			
M1. Baking powder, baking soda, yeast†	25	2.8±14.0	48.4±11.2
M2. Baking decoration	20	0±0	50.0±0
M3. Bread crumbs and batter mixes	241	7.3±20.7	27.7±14
M5. Cocoa powder	5	0±0	50.0±0
M7. Chewing gum	3	0±0	50.0±0
M8. Salad and potato toppers	23	33.9±35.4	63.0±17.1
M9. Salt, salt substitutes†	166	8.5±15.4	41.6±15.3
M10. Spices and herbs without salt	36	23.6±26.3	61.8±13.2
M11. Coconut milk	19	0±0	25.0±0
M12. Dried coconut	14	55±29.8	38.8±7.5
**Category M Total**	**552**	**10.2±22**	**37.8±17.8**
**N. Combination Dishes**			
N1. Combination dishes	529	19.7±29.8	34.4±20.1
N2. Burritos, pizzas, sandwiches, meat pie, etc. †	408	14.9±25.2	28.8±15.8
N3. Hors d'oeuvres	124	31.5±35	32.7±14.8
**Category N Total**	**1,061**	**19.2±29.2**	**32.0±18.2**
**O. Nuts & Seeds**			
O1. Nuts and seeds (not used for snacks)§	140	92.4±13	96.2±6.5
O2. Nut pastes and creams	7	32.9±35.5	30.0±12.5
O3. Nut butters	101	78.3±16.7	83.4±17.6
O4. Nut flours	4	100±0	75.0±28.9
**Category O Total**	**252**	**85.2±19.1**	**88.9±17.4**
**P. Potatoes**			
P1. French fries	65	70.0±0	76.5±17.1
P2. Mashed, stuffed, candied potatoes	37	39.7±35.2	35.0±17.2
P3. Fresh, canned, frozen potatoes	29	86.6±15.2	85.9±21.2
**Category P Total**	**131**	**65.1±26.3**	**66.9±27.2**
**Q. Salads**			
Q1. Salads	84	71±21.8	55.7±27.1
Q3. Pasta, potato or grain-based salad	20	43±37.3	45.6±28.8
**Category Q Total**	**104**	**65.6±27.6**	**53.8±27.6**
**R. Sauces & Dips**			
R1. Dipping sauces	158	3.3±10.3	29.9±14.3
R2. Dips and spreads	183	53.6±35	66.7±27.7
R3. Major main entrée sauce	225	32±33.9	39.6±22.7
R4. Minor main entrée sauce	264	24.5±32.3	43.1±24.2

TRA Category[*]	n	Nutritious food point (i.e., Step 1), means±SD	Final CFSS score, means±SD
R5. Major condiments[†,‡]	293	12.9±25.2	42.6±14.7
R6. Minor condiments[†,⁑]	121	7.5±21.5	53.6±11.1
**Category R Total**	**1,244**	**23.1±32.6**	**45.2±23**
**S. Snacks**			
S1. Chips, pretzels, etc.[†]	562	42.2±34.1	52.3±24.6
S2. Nuts or seeds (used as snacks)	252	69±21.2	69.8±24.2
S3. Meat or poultry sticks	31	50.0±0	31.7±14.7
**Category S Total**	**845**	**50.5±32.5**	**56.8±25.9**
**T. Soups**			
T1. All varieties of soups (includes broth)	475	9.2±17.1	26.2±8.7
**Category T Total**	**475**	**9.2±17.1**	**26.2±8.7**
**U. Sugars & Sweets**			
U1. Candies, confectionaries, chocolates	534	2.6±11.1	21.8±5.5
U3. Hard candies	20	0±0	41.3±12.2
U4. Baking candies	47	1.9±10.6	23.9±4.3
U5. Breath mints	2	0±0	50±0
U7. Confectioner's or icing sugar	3	0±0	50±0
U8. Honey, molasses, bread spreads	72	0.6±3.3	45.9±10.4
U9. Jams, jellies, fruit spreads	225	43.6±32.9	68.3±19.5
U10. Fruit leather	20	7±21.5	26.1±4
U11. Marshmallows	12	0±0	25.0±0
U12. Sugars[†,‡]	45	0±0	50.0±0
U14. Syrups used as toppings	52	4.8±16.7	52.4±8.4
U15. Syrups used as ingredients	20	3.5±15.7	49.3±11.4
**Category U Total**	**1,052**	**11.2±24.7**	**37.4±21.9**
**V. Vegetables**			
V1. Vegetables without sauce	407	78.5±13.3	86.6±13.1
V2. Vegetables with sauce	10	64.0±35.0	49.4±29.7
V3. Vegetables used for garnishing/flavouring[†,⁑]	27	71.1±5.8	85.6±2.9
V4. Chili pepper & green onion[†]	42	70.5±13.6	61.1±23.7
V5. Seaweed, dehydrated mushrooms	19	87.4±15.2	93.7±7.6
V6. Sprouts	1	100.0±0	100.0±0
V7. Vegetable juice and drink	58	0±0	38.4±12.6
V8. Olives	65	70.3±4.3	58.6±21.2
V9. Sun-dried tomatoes and other pickled or oil-packed vegetables	170	69.4±10.2	57.6±21.0
V10. Relish	15	68.0±20.1	81.2±14.7
V11. Vegetable paste	12	58.3±27.2	79.2±13.6
V12. Vegetable sauce or purée	34	35.0±35.5	63.8±23
**Category V Total**	**860**	**68±24.9**	**72.9±23.3**
**W. Foods for <4 years old[⁂]**			
W1. Cereals to be prepared[†]	41	45.9±35.2	57.1±27.2
W2. Ready-to-eat cereals and cereal bars[†]	9	67.8±6.7	41.9±1.7
W3. Cookies, biscuits, etc. [†]	46	31.1±33.9	61.1±19.3
W4. Strained meat, desserts, combination dishes[†]	89	1.7±8.4	33.9±13.4
W5. Combination dishes[†]	8	0±0	50.0±0
W6. Juices[†]	2	0±0	25.0±0
**Category W Total**	**195**	**20.9±31.7**	**46.2±21.8**
**OVERALL TOTAL**	**17,008**	**30.2±34.2**	**47.5±25.6**

Pre-packaged foods in Food Label Information and Price (FLIP) 2017 were used in the analyses (n=17,008). All values are presented as means±SD. The nutritious food points from Step 1 of the Canadian Food Scoring System (CFSS) represented the alignment of foods with the nutritious food recommendations in Canada's food guide (CFG) and Guideline 1 of Canada's Dietary Guidelines for Health Professionals and Policymakers (CDG) using existing labelling regulations and standards [1]. The nutritious food points ranged from 0 to 100, with higher points representing a greater presence of nutritious foods recommended by CFG. The final CFSS scores combined the nutritious food points from Step 1 and the deduction proportion from Step 2. The deduction proportion operationalized the

recommendations on nutrients-of-concern in CFG and Guideline 2 of CDG using the exemption criteria and nutrient thresholds of Canadian front-of-pack labelling regulations. The final CFSS scores ranged from 10-100, with higher scores representing better alignment with the recommendations of CFG and CDG. *Health Canada's Table of Reference Amounts for Food [2] was used to define food categories. †Indicates categories with products that were missing values for saturated fat (n=217; 1.3% overall). ‡Indicates categories with products that were missing values for sugars (n=5; 0.03% overall). ‡Indicates categories with products that were missing values for sodium (n=10; 0.06% overall). **Although foods for <1-year-olds would be exempted from front-of-pack labelling regulations [3], all foods for <4-year-olds with a Nutrition Facts table were included as only the minimum age for consumption (e.g., ≥6-month-olds), not maximum age for consumption, are indicated in these foods. Abbreviations: CDG, Canada's Dietary Guidelines for Health Professionals and Policymakers; CFG, Canada's food guide; CFSS, Canadian Food Scoring System; FLIP, Food Label Information and Price; TRA, Table of Reference Amounts for Food.

References:

1. Lee JJ, Mulligan C, L'Abbé MR. Development and validity testing of the Canadian Food Scoring System (CFSS), a nutrient profile model based on the recommendations of Canada's food guide 2019. Appl Physiol Nutr Metab. 2024. doi: 10.1139/apnm-2024-0034 %M 39013203.
2. Health Canada. Table of Reference Amounts for Food. 2016 [cited 2019 July 15]. Available from: https://www.canada.ca/en/health-canada/services/technical-documents-labelling-requirements/table-reference-amount-food-2016.html.
3. Government of Canada. Regulations Amending the Food and Drug Regulations (Nutrition Symbols, Other Labelling Provisions, Vitamin D and Hydrogenated Fats or Oils): SOR/2022-168. Ottawa: Government of Canada; 2022 [cited 2022 July 30]. Available from: https://canadagazette.gc.ca/rp-pr/p2/2022/2022-07-20/html/sor-dors168-eng.html.
