# Supplementary material for: Assessing the extent to which front-of-pack labelling regulations could support healthy eating among Canadians
Source: PLoS One. 2025 Oct 8;20(10):e0330720. doi: 10.1371/journal.pone.0330720 (PMC12507316; doi:10.1371/journal.pone.0330720)
Supplement: S1 Table — (PDF) [file pone.0330720.s001.zip › Lee_CND FOPL_S5.pdf]

**S5 Table.** Mean nutritious food points of pre-packaged foods by front-of-pack labelling (FOPL) regulation category.

| TRA Category* | n | FOPL category, means±SD | | | | | p-value† |
|---|---|---|---|---|---|---|---|
| | | No 'High in' nutrition symbol | | 'High in' nutrition symbol | | | |
| | | Exempted | <Thresholds | 1 nutrient | 2 nutrients | 3 nutrients | |
| A. Bakery Products | 2,511 | N/A | 25.6±34.2[a] | 16.0±28.8[b] | 10.8±25.1[c] | 2.9±9.2[c] | <0.001 |
| B. Beverages | 843 | N/A | 34.9±47.7[a] | 2.8±16.6[b] | 5.6±23.6[b] | 0±0[ab] | <0.001 |
| C. Cereals & Other Grains | 1,275 | N/A | 30.0±39.0 | 26.3±34.6 | 16.3±28.7 | N/A | 0.11 |
| D. Dairy Products & Substitutes | 1,492 | 92.1±15.2[a] | 45.3±21.0[b] | 43.1±27.4[b] | 47.6±20.9[b] | 82.1±12.2[a] | <0.001 |
| E. Desserts | 679 | N/A | 2.2±9.2[ab] | 3.4±13.3[a] | 0.2±1.8[b] | 0±0[ab] | <0.001 |
| F. Dessert Toppings & Fillings | 94 | N/A | 40.0±37.4[a] | 19.9±32.7[ab] | 0±0[b] | N/A | 0.04 |
| G. Eggs & Substitutes | 61 | 78.7±16.4[a] | 58.0±11.0[ab] | 50.0±0[b] | N/A | N/A | <0.001 |
| H. Fats & Oils | 652 | 30.6±21.8[a] | 15.3±16.8[b] | 10.4±15.5[b] | 20±14.4[b] | N/A | <0.001 |
| I. Seafood & Substitutes | 446 | 76.3±15.3[a] | 50.4±3.6[b] | 51.2±6.8[b] | 51.6±5.5[b] | 50.0±0[b] | <0.001 |
| J. Fruits & Fruit Juices | 1,045 | 83.6±14.4[a] | 39.7±35.6[b] | 16.6±30.2[c] | 30.0±37.4[bc] | N/A | <0.001 |
| K. Legumes | 187 | N/A | 80.4±19.9[a] | 62.9±24.9[b] | N/A | N/A | <0.001 |
| L. Meats & Substitutes | 952 | 74.9±4.9[a] | 44.1±21.2[b] | 48.7±12.2[c] | 49.2±6.8[c] | 50.0±0[bc] | <0.001 |
| M. Miscellaneous | 552 | 2.9±7.3 | 13.0±23.7 | 9.8±21.7 | 6.5±19.7 | 0±0 | 0.06 |
| N. Combination Dishes | 1,061 | N/A | 31.5±34.3[a] | 19.1±29.2[b] | 13.7±24.5[c] | 20.9±33.3[abc] | <0.001 |
| O. Nuts & Seeds | 252 | 92.4±13.7[a] | 69.8±11.3[b] | 75.0±18.9[b] | 17.5±35.0[c] | N/A | <0.001 |
| P. Potatoes | 131 | 96.7±9.7[a] | 66.7±14.9[b] | 48.1±33.0[c] | 57.6±27.5[bc] | N/A | <0.001 |
| Q. Salads | 104 | N/A | 80.3±20.4[a] | 60.5±24.5[b] | 54.0±33.3[b] | N/A | <0.001 |
| R. Sauces & Dips | 1,244 | N/A | 28.4±36.2[a] | 20.6±29.6[b] | 9.7±20.2[c] | 18.8±25.9[bc] | <0.001 |
| S. Snacks | 845 | 70.0±17.0[a] | 55.4±30.8[a] | 45.1±34.3[b] | 40.3±30.8[b] | 32.0±44.4[ab] | <0.001 |
| T. Soups | 475 | N/A | 6.2±9.4 | 10.1±16.2 | 7.8±19.9 | 7.5±20.5 | 0.45 |
| U. Sugars & Sweets | 1,052 | 1.8±10.6[a] | 39.3±33.7[b] | 4.4±16.3[a] | 3.1±11.8[a] | N/A | <0.001 |
| V. Vegetables | 860 | 81.6±21.9[a] | 63.9±21.7[b] | 58.9±26.5[b] | 72.5±12.8[ab] | N/A | <0.001 |
| W. Foods for <4-year-olds‡ | 195 | N/A | 26.9±34.1[a] | 14.7±28.0[b] | N/A | N/A | 0.007 |
| **OVERALL TOTAL** | **17,008** | **61.0±36.9[a]** | **36.9±35.6[b]** | **23.4±30.6[c]** | **18.8±26.6[d]** | **17.9±28.4[cd]** | **<0.001** |

Pre-packaged foods in Food Label Information and Price (FLIP) 2017 were used in the analyses (n=17,008). All values are presented as means±SD. According to Canadian FOPL regulations [1], foods would not display a 'High in' nutrition symbol if they meet the exemption criteria (i.e., "Exempted") or have nutrient levels below thresholds for all 3 nutrients-of-concern (i.e., "<Thresholds"). Foods would display a 'High in' nutrition symbol (i.e., "1-3 Nutrients") for meeting and/or exceeding thresholds for nutrient(s)-of-concern (saturated fat, total sugars, or sodium). The nutritious food points from Step 1 of the Canadian Food Scoring System represented the alignment of foods with the nutritious food recommendations in Canada's food guide (CFG) and Guideline 1 of Canada's Dietary Guidelines for Health

Professionals and Policymakers (CDG) using existing labelling regulations and standards [3]. The nutritious food points ranged from 0 to 100, with higher points representing a greater presence of nutritious foods recommended by CFG. [*]Health Canada's TRA [2] was used to define food categories. [†]Indicates categories with products that were missing values for saturated fat (n=217; 1.3% overall). [⁑]Indicates categories with products that were missing values for sugars (n=5; 0.03% overall). [‡]Indicates categories with products that were missing values for sodium (n=10; 0.06% overall). [⁂]Although foods for <1-year-olds would be exempted from front-of-pack labelling regulations, all foods for <4-year-olds with a Nutrition Facts table were included as only the minimum age for consumption (e.g., ≥6-month-olds), not maximum age for consumption, are indicated in these foods. Abbreviations: CDG, Canada's Dietary Guidelines for Health Professionals and Policymakers; CFG, Canada's food guide; FLIP, Food Label Information and Price; FOPL, front-of-pack labelling; TRA, Table of Reference Amounts for Food.

**References:**

1. Government of Canada. Regulations Amending the Food and Drug Regulations (Nutrition Symbols, Other Labelling Provisions, Vitamin D and Hydrogenated Fats or Oils): SOR/2022-168. Ottawa: Government of Canada; 2022 [cited 2022 July 30]. Available from: https://canadagazette.gc.ca/rp-pr/p2/2022/2022-07-20/html/sor-dors168-eng.html.
2. Health Canada. Table of Reference Amounts for Food. 2016 [cited 2019 July 15]. Available from: https://www.canada.ca/en/health-canada/services/technical-documents-labelling-requirements/table-reference-amount-food-2016.html.
3. Lee JJ, Mulligan C, L'Abbé MR. Development and validity testing of the Canadian Food Scoring System (CFSS), a nutrient profile model based on the recommendations of Canada's food guide 2019. Appl Physiol Nutr Metab. 2024. doi: 10.1139/apnm-2024-0034 %M 39013203.
