# Supplementary material for: Assessing the extent to which front-of-pack labelling regulations could support healthy eating among Canadians
Source: PLoS One. 2025 Oct 8;20(10):e0330720. doi: 10.1371/journal.pone.0330720 (PMC12507316; doi:10.1371/journal.pone.0330720)
Supplement: S1 Table — (PDF) [file pone.0330720.s001.zip › Lee_CND FOPL_S6.pdf]

S6 Table. Mean Canadian Food Scoring System scores of pre-packaged foods by front-of-pack labelling regulation category.

TRA Category*	n	FOPL category, means±SD					p-value†
		No 'High in' nutrition symbol		'High in' nutrition symbol			
		Exempted	<Thresholds	1 nutrient	2 nutrients	3 nutrients	
A. Bakery Products	2,511	N/A	62.8±17.1[a]	29.0±7.2[b]	19.4±4.4[c]	10.3±0.9[d]	<0.001
B. Beverages	843	N/A	67.5±23.9[a]	25.7±4.1[b]	18.5±4.1[b]	10.0±0[b]	<0.001
C. Cereals & Other Grains	1,275	N/A	65.0±19.5[a]	31.6±8.6[b]	20.3±5.0[c]	N/A	<0.001
D. Dairy Products & Substitutes	1,492	96.0±7.6[a]	72.6±10.5[b]	35.8±6.9[c]	25.8±3.7[d]	18.2±1.2[d]	<0.001
E. Desserts	679	N/A	51.1±4.6[a]	25.8±3.3[b]	17.5±0.3[c]	10.0±0[d]	<0.001
F. Dessert Toppings & Fillings	94	N/A	70.0±18.7[a]	30.0±8.2[b]	17.5±0[c]	N/A	<0.001
G. Eggs & Substitutes	61	89.3±8.2[a]	79.0±5.5[a]	37.5±0[b]	N/A	N/A	<0.001
H. Fats & Oils	652	65.3±10.9[a]	57.6±8.4[b]	27.6±3.9[c]	21±2.5[d]	N/A	<0.001
I. Seafood & Substitutes	446	88.1±7.7[a]	75.2±1.8[b]	37.8±1.7[c]	26.5±1.0[d]	15±0[e]	<0.001
J. Fruits & Fruit Juices	1,045	91.8±7.2[a]	69.8±17.8[b]	29.2±7.6[c]	22.8±6.5[c]	N/A	<0.001
K. Legumes	187	N/A	90.2±10.0[a]	40.7±6.2[b]	N/A	N/A	<0.001
L. Meats & Substitutes	952	87.4±2.5[a]	72.1±10.6[b]	37.2±3.1[c]	26.1±1.2[d]	15.0±0[e]	<0.001
M. Miscellaneous	552	51.4±3.6[a]	56.5±11.9[b]	27.4±5.4[c]	18.6±3.5[d]	10.0±0[e]	<0.001
N. Combination Dishes	1,061	N/A	65.8±17.2[a]	29.8±7.3[b]	19.9±4.3[c]	12.1±3.3[d]	<0.001
O. Nuts & Seeds	252	96.2±6.8[a]	84.9±5.7[b]	43.8±4.7[c]	20.6±6.1[d]	N/A	<0.001
P. Potatoes	131	98.3±4.9[a]	83.4±7.5[b]	37.0±8.2[c]	27.6±4.8[d]	N/A	<0.001
Q. Salads	104	N/A	90.1±10.2[a]	40.1±6.1[b]	27.0±5.8[c]	N/A	<0.001
R. Sauces & Dips	1,244	N/A	64.2±18.1[a]	30.2±7.4[b]	19.2±3.5[c]	11.9±2.6[c]	<0.001
S. Snacks	845	85.0±8.5[a]	77.7±15.4[b]	36.3±8.6[c]	24.6±5.4[d]	13.2±4.4[d]	<0.001
T. Soups	475	N/A	53.1±4.7[a]	27.5±4.0[b]	18.9±3.5[c]	10.8±2.1[d]	<0.001
U. Sugars & Sweets	1,052	50.9±5.3[a]	69.7±16.9[b]	26.1±4.1[c]	18.0±2.1[d]	N/A	<0.001
V. Vegetables	860	90.8±11[a]	81.9±10.9[b]	39.7±6.6[c]	30.2±2.2[d]	N/A	<0.001
W. Foods for <4-year-olds‡	195	N/A	63.5±17.1[a]	28.7±7.0[b]	N/A	N/A	<0.001
**OVERALL TOTAL**	**17,008**	**80.5±18.5[a]**	**68.5±17.8[b]**	**30.9±7.6[c]**	**20.8±4.7[d]**	**11.8±2.8[e]**	**<0.001**

Pre-packaged foods in Food Label Information and Price (FLIP) 2017 were used in the analyses (n=17,008). All values are presented as means±SD. According to Canadian FOPL regulations [1], foods would not display a 'High in' nutrition symbol if they meet the exemption criteria (i.e., "Exempted") or have nutrient levels below thresholds for all 3 nutrients-of-concern (i.e., "<Thresholds"). Foods would display a 'High in' nutrition symbol (i.e., "1-3 Nutrients") for meeting and/or exceeding thresholds for nutrient(s)-of-concern (saturated fat, total sugars, or sodium). The CFSS assessed the alignment of individual foods with the recommendations of Canada's food guide (CFG) and Canada's Dietary Guidelines for Health Professionals and Policymakers (CDG) using existing labelling regulations and standards

[3]. The final CFSS scores ranged from 10-100, with higher scores representing better alignment with the recommendations of CFG and CDG. *Health Canada's Table of Reference Amounts for Food (TRA) [2] was used to define food categories. †Means in a row with different letters represent significant differences ($p<0.05$) by one-factor ANOVA with Tukey-Kramer's *post hoc* test to account for multiple comparisons. ‡Although foods for <1-year-olds would be exempted from front-of-pack labelling regulations, all foods for <4-year-olds with a Nutrition Facts table were included as only the minimum age for consumption (e.g., ≥6-month-olds), not maximum age for consumption, are indicated in these foods. Abbreviations: CDG, Canada's Dietary Guidelines for Health Professionals and Policymakers; CFG, Canada's food guide; CFSS, Canadian Food Scoring System; FOPL, front-of-pack labelling; TRA, Table of Reference Amounts for Food.

References:

1. Government of Canada. Regulations Amending the Food and Drug Regulations (Nutrition Symbols, Other Labelling Provisions, Vitamin D and Hydrogenated Fats or Oils): SOR/2022-168. Ottawa: Government of Canada; 2022 [cited 2022 July 30]. Available from: https://canadagazette.gc.ca/rp-pr/p2/2022/2022-07-20/html/sor-dors168-eng.html.
2. Health Canada. Table of Reference Amounts for Food. 2016 [cited 2019 July 15]. Available from: https://www.canada.ca/en/health-canada/services/technical-documents-labelling-requirements/table-reference-amount-food-2016.html.
3. Lee JJ, Mulligan C, L'Abbé MR. Development and validity testing of the Canadian Food Scoring System (CFSS), a nutrient profile model based on the recommendations of Canada's food guide 2019. Appl Physiol Nutr Metab. 2024. doi: 10.1139/apnm-2024-0034 %M 39013203.
