# Supplementary material for: Assessing the extent to which front-of-pack labelling regulations could support healthy eating among Canadians
Source: PLoS One. 2025 Oct 8;20(10):e0330720. doi: 10.1371/journal.pone.0330720 (PMC12507316; doi:10.1371/journal.pone.0330720)
Supplement: S1 Table — (PDF) [file pone.0330720.s001.zip › Lee_CND FOPL_S1.pdf]

Assessing the extent to which front-of-pack labelling regulations could support healthy eating among Canadians

Lee JJ, Mulligan C, Jeong H, L'Abbe MR

S1 Table. Number and proportion of pre-packaged foods classified according to front-of-pack labelling (FOPL) regulations.

TRA Category[*]	n	'High in' nutrition symbol, n (%)				No 'High in' nutrition symbol, n (%)		
		1 Nutrient	2 Nutrients	3 Nutrients	Total	Exempted	<Thresholds	Total
**A. Bakery Products**								
A1. Bread, excluding sweet quick-type	289	108 (37.4%)	3 (1.0%)	0	111 (38.4%)	0	178 (61.6%)	178 (61.6%)
A2. Tea biscuits, scones, rolls, buns, etc.	235	74 (31.5%)	7 (3.0%)	0	81 (34.5%)	0	154 (65.5%)	154 (65.5%)
A3. Bagels, naan, flat bread	80	54 (67.5%)	1 (1.3%)	0	55 (68.8%)	0	25 (31.3%)	25 (31.3%)
A4. Brownies	29	13 (44.8%)	7 (24.1%)	0	20 (69.0%)	0	9 (31.0%)	9 (31.0%)
A5. Heavy weight cake	64	0	46 (71.9%)	18 (28.1%)	64 (100.0%)	0	0	0
A6. Medium weight cake	101	20 (19.8%)	77 (76.2%)	4 (4.0%)	101 (100.0%)	0	0	0
A7. Light weight cake	5	4 (80.0%)	1 (20.0%)	0	5 (100.0%)	0	0	0
A8. Coffee cakes, doughnuts, sweet quick-type breads, etc.	82	46 (56.1%)	17 (20.7%)	3 (3.7%)	66 (80.5%)	0	16 (19.5%)	16 (19.5%)
A9. Muffins	34	3 (8.8%)	22 (64.7%)	8 (23.5%)	33 (97.1%)	0	1 (2.9%)	1 (2.9%)
A10. Cookies[†]	501	187 (37.3%)	224 (44.7%)	0	411 (82.0%)	0	90 (18.0%)	90 (18.0%)
A11. Accompaniment crackers	254	42 (16.5%)	2 (0.8%)	0	44 (17.3%)	0	210 (82.7%)	210 (82.7%)
A12. Snack crackers	66	40 (60.6%)	4 (6.1%)	0	44 (66.7%)	0	22 (33.3%)	22 (33.3%)
A13. Dry breads	87	28 (32.2%)	5 (5.7%)	0	33 (37.9%)	0	54 (62.1%)	54 (62.1%)
A14. Toaster pastries	11	6 (54.5%)	0	0	6 (54.5%)	0	5 (45.5%)	5 (45.5%)
A15. Ice cream cones	20	0	0	0	0	0	20 (100.0%)	20 (100.0%)
A16. Croutons	37	2 (5.4%)	0	0	2 (5.4%)	0	35 (94.6%)	35 (94.6%)
A17. French toast, pancakes, waffles	54	44 (81.5%)	1 (1.9%)	1 (1.9%)	46 (85.2%)	0	8 (14.8%)	8 (14.8%)
A18. Grain-based bars with filling	105	20 (19.0%)	13 (12.4%)	0	33 (31.4%)	0	72 (68.6%)	72 (68.6%)
A19. Grain-based bars without filling	94	25 (26.6%)	7 (7.4%)	0	32 (34.0%)	0	62 (66.0%)	62 (66.0%)
A20. Energy and protein bars	205	97 (47.3%)	26 (12.7%)	1 (0.5%)	124 (60.5%)	0	81 (39.5%)	81 (39.5%)
A21. Rice and corn cakes	35	11 (31.4%)	0	0	11 (31.4%)	0	24 (68.6%)	24 (68.6%)
A22. Pies, pastries, etc.	79	9 (11.4%)	60 (75.9%)	10 (12.7%)	79 (100.0%)	0	0	0
A23. Pie crust	18	18 (100.0%)	0	0	18 (100.0%)	0	0	0
A24. Pizza crust	13	3 (23.1%)	0	0	3 (23.1%)	0	10 (76.9%)	10 (76.9%)
A25. Taco shell	13	5 (38.5%)	2 (15.4%)	0	7 (53.8%)	0	6 (46.2%)	6 (46.2%)
**Category A Total**	**2,511**	**859 (34.2%)**	**525 (20.9%)**	**45 (1.8%)**	**1,429 (56.9%)**	**0**	**1,082 (43.1%)**	**1,082 (43.1%)**
**B. Beverages**								
B1. Carbonated and non-carbonated beverages[†]	713	357 (50.1%)	13 (1.8%)	1 (0.1%)	371 (52.0%)	0	342 (48.0%)	342 (48.0%)
B3. Coffee[†,⁑,‡]	28	5 (17.9%)	1 (3.6%)	0	6 (21.4%)	0	22 (78.6%)	22 (78.6%)
B4. Tea	70	6 (8.6%)	0	0	6 (8.6%)	0	64 (91.4%)	64 (91.4%)
B5. Cocoa and hot chocolate beverages	32	21 (65.6%)	4 (12.5%)	0	25 (78.1%)	0	7 (21.9%)	7 (21.9%)
**Category B Total**	**843**	**389 (46.1%)**	**18 (2.1%)**	**1 (0.1%)**	**408 (48.4%)**	**0**	**435 (51.6%)**	**435 (51.6%)**
**C. Cereals, Other Grain Products and Substitutes**								
C1. Hot breakfast cereals	118	6 (5.1%)	0	0	6 (5.1%)	0	112 (94.9%)	112 (94.9%)
C2. Ready-to-eat cereals, puffed and uncoated	4	0	0	0	0	0	4 (100.0%)	4 (100.0%)

TRA Category*	n	'High in' nutrition symbol, n (%)				No 'High in' nutrition symbol, n (%)		
		1 Nutrient	2 Nutrients	3 Nutrients	Total	Exempted	<Thresholds	Total
C3. Ready-to-eat cereals, puffed and coated without fruit or nuts	88	28 (31.8%)	2 (2.3%)	0	30 (34.1%)	0	58 (65.9%)	58 (65.9%)
C4. Ready-to-eat cereals, fruit and nut, granola type	169	35 (20.7%)	4 (2.4%)	0	39 (23.1%)	0	130 (76.9%)	130 (76.9%)
C5. Bran and wheat germ, milled flax, etc.	38	5 (13.2%)	0	0	5 (13.2%)	0	33 (86.8%)	33 (86.8%)
C6. Flours and cornmeal	66	2 (3.0%)	0	0	2 (3.0%)	0	64 (97.0%)	64 (97.0%)
C7. Grains	279	82 (29.4%)	1 (0.4%)	0	83 (29.7%)	0	196 (70.3%)	196 (70.3%)
C8. Pastas	492	19 (3.9%)	17 (3.5%)	0	36 (7.3%)	0	456 (92.7%)	456 (92.7%)
C10. Starch	11	1 (9.1%)	0	0	1 (9.1%)	0	10 (90.9%)	10 (90.9%)
C11. Stuffing	10	9 (90.0%)	0	0	9 (90.0%)	0	1 (10.0%)	1 (10.0%)
**Category C Total**	**1,275**	**187 (14.7%)**	**24 (1.9%)**	**0**	**211 (16.5%)**	**0**	**1,064 (83.5%)**	**1,064 (83.5%)**
**D. Dairy Products & Substitutes**								
D1. Cheese	568	81 (14.3%)	47 (8.3%)	0	128 (22.5%)	0	440 (77.5%)	440 (77.5%)
D2. Cottage cheese	21	2 (9.5%)	0	0	2 (9.5%)	0	19 (90.5%)	19 (90.5%)
D3. Cheese used as ingredient	14	7 (50.0%)	0	0	7 (50.0%)	0	7 (50.0%)	7 (50.0%)
D4. Hard cheese	44	9 (20.5%)	6 (13.6%)	0	15 (34.1%)	0	29 (65.9%)	29 (65.9%)
D5. Quark	110	10 (9.1%)	35 (31.8%)	0	45 (40.9%)	0	65 (59.1%)	65 (59.1%)
D6. Cream and cream substitutes	41	0	0	0	0	14 (34.1%)	27 (65.9%)	41 (100.0%)
D7. Powder cream and cream substitutes	9	2 (22.2%)	0	0	2 (22.2%)	0	7 (77.8%)	7 (77.8%)
D8. Aerosol/whipped cream and cream substitutes	22	10 (45.5%)	0	0	10 (45.5%)	0	12 (54.5%)	12 (54.5%)
D10. Evaporated/condensed milk	17	7 (41.2%)	0	0	7 (41.2%)	9 (52.9%)	1 (5.9%)	10 (58.8%)
D11. Milk, buttermilk, milk-based drinks, plant-based milk substitutes	200	47 (23.5%)	1 (0.5%)	6 (3.0%)	54 (27.0%)	59 (29.5%)	87 (43.5%)	146 (73.0%)
D12. Fermented dairy drinks	59	47 (79.7%)	1 (1.7%)	0	48 (81.4%)	0	11 (18.6%)	11 (18.6%)
D13. Shakes and smoothies	25	11 (44.0%)	6 (24.0%)	1 (4.0%)	18 (72.0%)	0	7 (28.0%)	7 (28.0%)
D14. Sour cream	24	13 (54.2%)	0	0	13 (54.2%)	0	11 (45.8%)	11 (45.8%)
D15. Yogurt	338	190 (56.2%)	52 (15.4%)	0	242 (71.6%)	0	96 (28.4%)	96 (28.4%)
**Category D Total**	**1,492**	**436 (29.2%)**	**148 (9.9%)**	**7 (0.5%)**	**591 (39.6%)**	**82 (5.5%)**	**819 (54.9%)**	**901 (60.4%)**
**E. Desserts**								
E1. Ice cream, frozen yogurt, sherbet, etc. in tubs	277	57 (20.6%)	218 (78.7%)	2 (0.7%)	277 (100.0%)	0	0	0
E2 Ice cream, frozen yogurt, sherbet, etc. as cakes, cones	61	13 (21.3%)	43 (70.5%)	1 (1.6%)	57 (93.4%)	0	4 (6.6%)	4 (6.6%)
E3 Ice cream, frozen yogurt, sherbet, etc. as pops, bars	153	49 (32.0%)	58 (37.9%)	0	107 (69.9%)	0	46 (30.1%)	46 (30.1%)
E4. Sundaes	8	0	8 (100.0%)	0	8 (100.0%)	0	0	0
E5. Custard, gelatin, pudding	180	86 (47.8%)	62 (34.4%)	4 (2.2%)	152 (84.4%)	0	28 (15.6%)	28 (15.6%)
**Category E Total**	**679**	**205 (30.2%)**	**389 (57.3%)**	**7 (1.0%)**	**601 (88.5%)**	**0**	**78 (11.5%)**	**78 (11.5%)**
**F. Dessert Toppings & Fillings**								
F1. Dessert toppings	29	22 (75.9%)	4 (13.8%)	0	26 (89.7%)	0	3 (10.3%)	3 (10.3%)
F2. Cake frostings[‡]	35	31 (88.6%)	4 (11.4%)	0	35 (100.0%)	0	0	0
F3. Pie fillings	30	25 (83.3%)	1 (3.3%)	0	26 (86.7%)	0	4 (13.3%)	4 (13.3%)

TRA Category*	n	'High in' nutrition symbol, n (%)				No 'High in' nutrition symbol, n (%)		
		1 Nutrient	2 Nutrients	3 Nutrients	Total	Exempted	<Thresholds	Total
**Category F Total**	**94**	**78 (83.0%)**	**9 (9.6%)**	**0**	**87 (92.5%)**	**0**	**7 (7.4%)**	**7 (7.4%)**
**G. Eggs & Substitutes**								
G1. Egg mixtures	5	2 (40.0%)	0	0	2 (40.0%)	0	3 (60.0%)	3 (60.0%)
G2. Eggs‡	56	2 (3.6%)	0	0	2 (3.6%)	52 (92.9%)	2 (3.6%)	54 (96.4%)
**Category G Total**	**61**	**4 (6.6%)**	**0**	**0**	**4 (6.6%)**	**52 (85.2%)**	**5 (8.2%)**	**57 (93.4%)**
**H. Fats & Oils**								
H1. Butter, margarine, lard, etc.	112	0	0	0	0	112 (100.0%)	0	112 (100.0%)
H2. Vegetable oil	166	0	0	0	0	166 (100.0%)	0	166 (100.0%)
H4. Dressings for salad	283	134 (47.3%)	27 (9.5%)	0	161 (56.9%)	0	122 (43.1%)	122 (43.1%)
H5. Mayonnaise and mayonnaise-type dressing	68	0	0	0	0	0	68 (100.0%)	68 (100.0%)
H6. Spray oil	23	0	0	0	0	23 (100.0%)	0	23 (100.0%)
**Category H Total**	**652**	**134 (20.6%)**	**27 (4.1%)**	**0**	**161 (24.7%)**	**301 (46.2%)**	**190 (29.1%)**	**491 (75.3%)**
**I. Seafood & Substitutes**								
I1. Anchovies, caviar	9	6 (66.7%)	0	0	6 (66.7%)	0	3 (33.3%)	3 (33.3%)
I2. Marine and freshwater animals with sauce	50	31 (62.0%)	10 (20.0%)	1 (2.0%)	42 (84.0%)	0	8 (16.0%)	8 (16.0%)
I3. Marine and freshwater animals without sauce	191	92 (48.2%)	15 (7.9%)	0	107 (56.0%)	36 (18.8%)	48 (25.1%)	84 (44.0%)
I4. Canned marine and freshwater animals	147	27 (18.4%)	0	0	27 (18.4%)	12 (8.2%)	108 (73.5%)	120 (81.6%)
I5. Smoked/pickled marine and freshwater animals	49	33 (67.3%)	0	0	33 (67.3%)	0	16 (32.7%)	16 (32.7%)
**Category I Total**	**446**	**189 (42.4%)**	**25 (5.6%)**	**1 (0.2%)**	**215 (48.2%)**	**48 (10.8%)**	**183 (41.0%)**	**231 (51.8%)**
**J. Fruits & Fruit Juices**								
J1. Fruits (fresh, frozen, canned, coated, and uncoated)	186	88 (47.3%)	0	0	88 (47.3%)	60 (32.3%)	38 (20.4%)	98 (52.7%)
J2. Berries	15	0	0	0	0	15 (100.0%)	0	15 (100.0%)
J3. Melons	5	0	0	0	0	5 (100.0%)	0	5 (100.0%)
J4. Avocados	1	0	0	0	0	1 (100.0%)	0	1 (100.0%)
J5. Cranberries, lemons, limes	3	0	0	0	0	3 (100.0%)	0	3 (100.0%)
J6. Fruit sauces and purees	65	19 (29.2%)	0	0	19 (29.2%)	16 (24.6%)	30 (46.2%)	46 (70.8%)
J7. Dried fruits	131	44 (33.6%)	3 (2.3%)	0	47 (35.9%)	70 (53.4%)	14 (10.7%)	84 (64.1%)
J8. Candied/pickled fruits	21	21 (100.0%)	0	0	21 (100.0%)	0	0	0
J9. Fruits for garnish	5	0	0	0	0	0	5 (100.0%)	5 (100.0%)
J11. Juices, nectars, fruit drinks	603	544 (90.2%)	4 (0.7%)	0	548 (90.9%)	0	55 (9.1%)	55 (9.1%)
J12. Fruit juices used as ingredients	10	0	0	0	0	0	10 (100.0%)	10 (100.0%)
**Category J Total**	**1,045**	**716 (68.5%)**	**7 (0.7%)**	**0**	**723 (69.2%)**	**170 (16.3%)**	**152 (14.5%)**	**322 (30.8%)**
**K. Legumes**								
K1. Tofu or tempeh	23	6 (26.1%)	0	0	6 (26.1%)	0	17 (73.9%)	17 (73.9%)
K2. Beans, lentils, etc.	164	18 (11.0%)	0	0	18 (11.0%)	0	146 (89.0%)	146 (89.0%)
**Category K Total**	**187**	**24 (12.8%)**	**0**	**0**	**24 (12.8%)**	**0**	**163 (87.2%)**	**163 (87.2%)**
**L. Meats & Substitutes**								
L1. Pork rinds and bacon	41	19 (46.3%)	19 (46.3%)	0	38 (92.7%)	0	3 (7.3%)	3 (7.3%)

TRA Category[*]	n	'High in' nutrition symbol, n (%)				No 'High in' nutrition symbol, n (%)		
		1 Nutrient	2 Nutrients	3 Nutrients	Total	Exempted	<Thresholds	Total
L2. Beef, pork and poultry breakfast strips	6	3 (50.0%)	1 (16.7%)	0	4 (66.7%)	0	2 (33.3%)	2 (33.3%)
L3. Dried meat and poultry	96	16 (16.7%)	80 (83.3%)	0	96 (100.0%)	0	0	0
L4. Luncheon meats	85	57 (67.1%)	23 (27.1%)	0	80 (94.1%)	0	5 (5.9%)	5 (5.9%)
L5. Sausage products	160	38 (23.8%)	116 (72.5%)	0	154 (96.3%)	0	6 (3.8%)	6 (3.8%)
L6. Cust of meat & poultry without sauce	125	71 (56.8%)	32 (25.6%)	0	103 (82.4%)	15 (12.0%)	7 (5.6%)	22 (17.6%)
L7. Patties, ground meat with and without breading	214	87 (40.7%)	71 (33.2%)	0	158 (73.8%)	20 (9.3%)	36 (16.8%)	56 (26.2%)
L8. Cured meats	86	68 (79.1%)	15 (17.4%)	0	83 (96.5%)	0	3 (3.5%)	3 (3.5%)
L9. Canned meats	27	8 (29.6%)	14 (51.9%)	0	22 (81.5%)	0	5 (18.5%)	5 (18.5%)
L10. Meat and poultry with sauce	112	33 (29.5%)	62 (55.4%)	16 (14.3%)	111 (99.1%)	0	1 (0.9%)	1 (0.9%)
**Category L Total**	**952**	**400 (42.0%)**	**433 (45.5%)**	**16 (1.7%)**	**849 (89.2%)**	**35 (3.7%)**	**68 (7.1%)**	**103 (10.8%)**
**M. Miscellaneous**								
M1. Baking powder, baking soda, yeast[†]	25	3 (12.0%)	0	0	3 (12.0%)	0	22 (88.0%)	22 (88.0%)
M2. Baking decoration	20	0	0	0	0	0	20 (100.0%)	20 (100.0%)
M3. Breadcrumbs	241	129 (53.5%)	71 (29.5%)	9 (3.7%)	209 (86.7%)	0	32 (13.3%)	32 (13.3%)
M5. Cocoa powder	5	0	0	0	0	0	5 (100.0%)	5 (100.0%)
M7. Chewing gum	3	0	0	0	0	0	3 (100.0%)	3 (100.0%)
M8. Salad and potato toppers	23	2 (8.7%)	0	0	2 (8.7%)	0	21 (91.3%)	21 (91.3%)
M9. Salt, salt substitutes[†]	166	78 (47.0%)	0	0	78 (47.0%)	14 (8.4%)	74 (44.6%)	88 (53.0%)
M10. Spices and herbs without salt	36	0	0	0	0	0	36 (100.0%)	36 (100.0%)
M11. Coconut milk	19	19 (100.0%)	0	0	19 (100.0%)	0	0	0
M12. Dried coconut	14	14 (100.0%)	0	0	14 (100.0%)	0	0	0
**Category M Total**	**552**	**245 (44.4%)**	**71 (12.9%)**	**9 (1.6%)**	**325 (58.9%)**	**14 (2.5%)**	**213 (38.6%)**	**227 (41.1%)**
**N. Combination Dishes**								
N1. Combination dishes	529	258 (48.8%)	150 (28.4%)	8 (1.5%)	416 (78.6%)	0	113 (21.4%)	113 (21.4%)
N2. Burritos, pizzas, sandwiches, meat pie, etc. [†]	408	161 (39.5%)	194 (47.5%)	1 (0.2%)	356 (87.3%)	0	52 (12.7%)	52 (12.7%)
N3. Hors d'oeuvres	124	71 (57.3%)	39 (31.5%)	2 (1.6%)	112 (90.3%)	0	12 (9.7%)	12 (9.7%)
**Category N Total**	**1,061**	**490 (46.2%)**	**383 (36.1%)**	**11 (1.0%)**	**884 (83.3%)**	**0**	**177 (16.7%)**	**177 (16.7%)**
**O. Nuts & Seeds**								
O1. Nuts and seeds (not used for snacks)[‡]	140	0	0	0	0	140 (100.0%)	0	140 (100.0%)
O2. Nut pastes and creams	7	4 (57.1%)	3 (42.9%)	0	7 (100.0%)	0	0	0
O3. Nut butters	101	12 (11.9%)	1 (1.0%)	0	13 (12.9%)	37 (36.6%)	51 (50.5%)	88 (87.1%)
O4. Nut flours	4	2 (50.0%)	0	0	2 (50.0%)	0	2 (50.0%)	2 (50.0%)
**Category O Total**	**252**	**18 (7.1%)**	**4 (1.6%)**	**0**	**22 (8.7%)**	**177 (70.2%)**	**53 (21.0%)**	**230 (91.3%)**
**P. Potatoes**								
P1. French fries	65	13 (20.0%)	0	0	13 (20.0%)	0	52 (80.0%)	52 (80.0%)
P2. Mashed, stuffed, candied potatoes	37	14 (37.8%)	17 (45.9%)	0	31 (83.8%)	0	6 (16.2%)	6 (16.2%)

TRA Category*	n	'High in' nutrition symbol, n (%)				No 'High in' nutrition symbol, n (%)		
		1 Nutrient	2 Nutrients	3 Nutrients	Total	Exempted	<Thresholds	Total
P3. Fresh, canned, frozen potatoes	29	5 (17.2%)	0	0	5 (17.2%)	18 (62.1%)	6 (20.7%)	24 (82.8%)
**Category P Total**	**131**	**32 (24.4%)**	**17 (13.0%)**	**0**	**49 (37.4%)**	**18 (13.7%)**	**64 (48.9%)**	**82 (62.6%)**
**Q. Salads**								
Q1. Salads	84	36 (42.9%)	19 (22.6%)	0	55 (65.5%)	0	29 (34.5%)	29 (34.5%)
Q3. Pasta, potato or grain-based salad	20	8 (40.0%)	6 (30.0%)	0	14 (70.0%)	0	6 (30.0%)	6 (30.0%)
**Category Q Total**	**104**	**44 (42.3%)**	**25 (24.0%)**	**0**	**69 (66.3%)**	**0**	**35 (33.7%)**	**35 (33.7%)**
**R. Sauces & Dips**								
R1. Dipping sauces	158	67 (42.4%)	52 (32.9%)	0	119 (75.3%)	0	39 (24.7%)	39 (24.7%)
R2. Dips and spreads	183	47 (25.7%)	9 (4.9%)	0	56 (30.6%)	0	127 (69.4%)	127 (69.4%)
R3. Major main entrée sauce	225	131 (58.2%)	43 (19.1%)	2 (0.9%)	176 (78.2%)	0	49 (21.8%)	49 (21.8%)
R4. Minor main entrée sauce	264	117 (44.3%)	36 (13.6%)	6 (2.3%)	159 (60.2%)	0	105 (39.8%)	105 (39.8%)
R5. Major condiments[†,‡]	293	127 (43.3%)	6 (2.0%)	0	133 (45.4%)	0	160 (54.6%)	160 (54.6%)
R6. Minor condiments[†,‡]	121	1 (0.8%)	0	0	1 (0.8%)	0	120 (99.2%)	120 (99.2%)
**Category R Total**	**1,244**	**490 (39.4%)**	**146 (11.7%)**	**8 (0.6%)**	**644 (51.8%)**	**0**	**600 (48.2%)**	**600 (48.2%)**
**S. Snacks**								
S1. Chips, pretzels, etc.[†]	562	233 (41.5%)	51 (9.1%)	5 (0.9%)	289 (51.4%)	0	273 (48.6%)	273 (48.6%)
S2. Nuts or seeds (used as snacks)	252	67 (26.6%)	19 (7.5%)	0	86 (34.1%)	35 (13.9%)	131 (52.0%)	166 (65.9%)
S3. Meat or poultry sticks	31	2 (6.5%)	26 (83.9%)	0	28 (90.3%)	0	3 (9.7%)	3 (9.7%)
**Category S Total**	**845**	**302 (35.7%)**	**96 (11.4%)**	**5 (0.6%)**	**403 (47.7%)**	**35 (4.1%)**	**407 (48.2%)**	**442 (52.3%)**
**T. Soups**								
T1. All varieties of soups (includes broth)	475	310 (65.3%)	127 (26.7%)	12 (2.5%)	449 (94.5%)	0	26 (5.5%)	26 (5.5%)
**Category T Total**	**475**	**310 (65.3%)**	**127 (26.7%)**	**12 (2.5%)**	**449 (94.5%)**	**0**	**26 (5.5%)**	**26 (5.5%)**
**U. Sugars & Sweets**								
U1. Candies, confectionaries, chocolates	534	241 (45.1%)	286 (53.6%)	0	527 (98.7%)	0	7 (1.3%)	7 (1.3%)
U3. Hard candies	20	7 (35.0%)	0	0	7 (35.0%)	0	13 (65.0%)	13 (65.0%)
U4. Baking candies	47	37 (78.7%)	10 (21.3%)	0	47 (100.0%)	0	0	0
U5. Breath mints	2	0	0	0	0	0	2 (100.0%)	2 (100.0%)
U7. Confectioner's or icing sugar	3	0	0	0	0	3 (100.0%)	0	3 (100.0%)
U8. Honey, molasses, bread spreads	72	3 (4.2%)	7 (9.7%)	0	10 (13.9%)	58 (80.6%)	4 (5.6%)	62 (86.1%)
U9. Jams, jellies, fruit spreads	225	23 (10.2%)	0	0	23 (10.2%)	0	202 (89.8%)	202 (89.8%)
U10. Fruit leather	20	19 (95.0%)	1 (5.0%)	0	20 (100.0%)	0	0	0
U11. Marshmallows	12	12 (100.0%)	0	0	12 (100.0%)	0	0	0
U12. Sugars[†,‡]	45	0	0	0	0	45 (100.0%)	0	45 (100.0%)
U14. Syrups used as toppings	52	0	0	0	0	52 (100.0%)	0	52 (100.0%)
U15. Syrups used as ingredients	20	2 (10.0%)	0	0	2 (10.0%)	18 (90.0%)	0	18 (90.0%)
**Category U Total**	**1,052**	**344 (32.7%)**	**304 (28.9%)**	**0**	**648 (61.6%)**	**176 (16.7%)**	**228 (21.7%)**	**404 (38.4%)**
**V. Vegetables**								
V1. Vegetables without sauce	407	25 (6.1%)	0	0	25 (6.1%)	219 (53.8%)	163 (40.0%)	382 (93.9%)
V2. Vegetables with sauce	10	3 (30.0%)	4 (40.0%)	0	7 (70.0%)	0	3 (30.0%)	3 (30.0%)

TRA Category[*]	n	'High in' nutrition symbol, n (%)				No 'High in' nutrition symbol, n (%)		
		1 Nutrient	2 Nutrients	3 Nutrients	Total	Exempted	<Thresholds	Total
V3. Vegetables used for garnishing/flavouring[†,‡]	27	0	0	0	0	12 (44.4%)	15 (55.6%)	27 (100.0%)
V4. Chili pepper & green onion[†]	42	23 (54.8%)	1 (2.4%)	0	24 (57.1%)	3 (7.1%)	15 (35.7%)	18 (42.9%)
V5. Seaweed, dehydrated mushrooms	19	0	0	0	0	12 (63.2%)	7 (36.8%)	19 (100.0%)
V6. Sprouts	1	0	0	0	0	1 (100.0%)	0	1 (100.0%)
V7. Vegetable juice and drink	58	27 (46.6%)	0	0	27 (46.6%)	8 (13.8%)	23 (39.7%)	31 (53.4%)
V8. Olives	65	38 (58.5%)	2 (3.1%)	0	40 (61.5%)	0	25 (38.5%)	25 (38.5%)
V9. Sun-dried tomatoes and other pickled or oil-packed vegetables	170	108 (63.5%)	1 (0.6%)	0	109 (64.1%)	0	61 (35.9%)	61 (35.9%)
V10. Relish	15	1 (6.7%)	0	0	1 (6.7%)	0	14 (93.3%)	14 (93.3%)
V11. Vegetable paste	12	0	0	0	0	7 (58.3%)	5 (41.7%)	12 (100.0%)
V12. Vegetable sauce or purée	34	5 (14.7%)	0	0	5 (14.7%)	0	29 (85.3%)	29 (85.3%)
**Category V Total**	**860**	**230 (26.7%)**	**8 (0.9%)**	**0**	**238 (27.7%)**	**262 (30.5%)**	**360 (41.9%)**	**622 (72.3%)**
**W. Foods for <4 years old[⁑]**								
W1. Cereals to be prepared[†]	41	20 (48.8%)	0	0	20 (48.8%)	0	21 (51.2%)	21 (51.2%)
W2. Ready-to-eat cereals and cereal bars[†]	9	9 (100.0%)	0	0	9 (100.0%)	0	0	0
W3. Cookies, biscuits, etc. [†]	46	6 (13.0%)	0	0	6 (13.0%)	0	40 (87.0%)	40 (87.0%)
W4. Strained meat, desserts, combination dishes[†]	89	60 (67.4%)	0	0	60 (67.4%)	0	29 (32.6%)	29 (32.6%)
W5. Combination dishes[†]	8	0	0	0	0	0	8 (100.0%)	8 (100.0%)
W6. Juices[†]	2	2 (100.0%)	0	0	2 (100.0%)	0	0	0
**Category W Total**	**195**	**97 (49.7%)**	**0**	**0**	**97 (49.7%)**	**0**	**98 (50.3%)**	**98 (50.3%)**
**OVERALL TOTAL**	**17,008**	**6,223 (36.6%)**	**2,786 (16.4%)**	**122 (0.7%)**	**9,131 (53.7%)**	**1,370 (8.1%)**	**6,507 (38.3%)**	**7,877 (46.3%)**

All values are presented as n (%). Pre-packaged foods in Food Label Information and Price (FLIP) 2017 were used for analyses (n=17,008). Canadian FOPL regulations mandate pre-packaged foods to display a 'High in' nutrition symbol (i.e., "1-3 Nutrients") if they meet and/or exceed thresholds for nutrients-of-concern (saturated fat, total sugars, or sodium) [1]. Foods would not display a 'High in' nutrition symbol if they meet the exemption criteria (i.e., "Exempted") or have nutrient levels below thresholds for all 3 nutrients-of-concern (i.e., "<Thresholds"). [*]Health Canada's Table of Reference Amounts for Food (TRA) [2] was used to define food categories. [†]Indicates categories with products that were missing values for saturated fat (n=217; 1.3% overall). [‡]Indicates categories with products that were missing values for sugars (n=5; 0.03% overall). [‡]Indicates categories with products that were missing values for sodium (n=10; 0.06% overall). [⁑]Although foods for <1-year-olds would be exempted from FOPL regulations [1], all foods for <4-year-olds with a Nutrition Facts table were included as only the minimum age for consumption (e.g., ≥6-month-olds), not maximum age for consumption, are indicated in these foods. Abbreviations: FLIP, Food Label Information and Price; FOPL, front-of-pack labelling; TRA, Table of Reference Amounts for Food

References:

1. Government of Canada. Regulations Amending the Food and Drug Regulations (Nutrition Symbols, Other Labelling Provisions, Vitamin D and Hydrogenated Fats or Oils): SOR/2022-168. Ottawa: Government of Canada; 2022 [cited 2022 July 30]. Available from: https://canadagazette.gc.ca/rp-pr/p2/2022/2022-07-20/html/sor-dors168-eng.html.
2. Health Canada. Table of Reference Amounts for Food. 2016 [cited 2019 July 15]. Available from: https://www.canada.ca/en/health-canada/services/technical-documents-labelling-requirements/table-reference-amount-food-2016.html.
