# Supplementary material for: Assessing the extent to which front-of-pack labelling regulations could support healthy eating among Canadians
Source: PLoS One. 2025 Oct 8;20(10):e0330720. doi: 10.1371/journal.pone.0330720 (PMC12507316; doi:10.1371/journal.pone.0330720)
Supplement: S1 Table — (PDF) [file pone.0330720.s001.zip › Lee_CND FOPL_S2.pdf]

Assessing the extent to which front-of-pack labelling regulations could support healthy eating among Canadians

Lee JJ, Mulligan C, Jeong H, L'Abbe MR

S2 Table. Number and proportion of pre-packaged foods that would display a 'High in' nutrition symbol according to front-of-pack labelling regulations presented by the nutrient-of-concern type.

TRA Category[*]	n	'High in' nutrient-of-concern type, n(%) Saturated fat	Sugars (total)	Sodium
**A. Bakery Products**				
A1. Bread, excluding sweet quick-type	289	3 (1.0%)	1 (0.3%)	110 (38.1%)
A2. Tea biscuits, scones, rolls, buns, etc.	235	21 (8.9%)	1 (0.4%)	66 (28.1%)
A3. Bagels, naan, flat bread	80	1 (1.3%)	0	55 (68.8%)
A4. Brownies	29	11 (37.9%)	16 (55.2%)	0
A5. Heavy weight cake	64	63 (98.4%)	64 (100.0%)	19 (29.7%)
A6. Medium weight cake	101	87 (86.1%)	91 (90.1%)	8 (7.9%)
A7. Light weight cake	5	0	5 (100.0%)	1 (20.0%)
A8. Coffee cakes, doughnuts, sweet quick-type breads, etc.	82	38 (46.3%)	44 (53.7%)	7 (8.5%)
A9. Muffins	34	30 (88.2%)	33 (97.1%)	8 (23.5%)
A10. Cookies[†]	501	348 (69.5%)	287 (57.3%)	0
A11. Accompaniment crackers	254	21 (8.3%)	0	25 (9.8%)
A12. Snack crackers	66	8 (12.1%)	2 (3.0%)	38 (57.6%)
A13. Dry breads	87	9 (10.3%)	3 (3.4%)	26 (29.9%)
A14. Toaster pastries	11	1 (9.1%)	5 (45.5%)	0
A15. Ice cream cones	20	0	0	0
A16. Croutons	37	1 (2.7%)	0	1 (2.7%)
A17. French toast, pancakes, waffles	54	2 (3.7%)	2 (3.7%)	45 (83.3%)
A18. Grain-based bars with filling	105	22 (21.0%)	24 (22.9%)	0
A19. Grain-based bars without filling	94	10 (10.6%)	28 (29.8%)	1 (1.1%)
A20. Energy and protein bars	205	95 (46.3%)	56 (27.3%)	1 (0.5%)
A21. Rice and corn cakes	35	2 (5.7%)	0	9 (25.7%)
A22. Pies, pastries, etc.	79	74 (93.7%)	73 (92.4%)	12 (15.2%)
A23. Pie crust	18	18 (100.0%)	0	0
A24. Pizza crust	13	0	0	3 (23.1%)
A25. Taco shell	13	6 (46.2%)	0	3 (23.1%)
**Category A Total**	**2,511**	**871 (34.7%)**	**735 (29.3%)**	**438 (17.4%)**
**B. Beverages**				
B1. Carbonated and non-carbonated beverages[†]	713	11 (1.5%)	362 (50.8%)	13 (1.8%)
B3. Coffee[†,⁑,‡]	28	5 (17.9%)	2 (7.1%)	0
B4. Tea	70	0	6 (8.6%)	0
B5. Cocoa and hot chocolate beverages	32	5 (15.6%)	24 (75.0%)	0
**Category B Total**	**843**	**21 (2.5%)**	**394 (46.7%)**	**13 (1.5%)**
**C. Cereals & Other Grains**				
C1. Hot breakfast cereals	118	1 (0.8%)	5 (4.2%)	0
C2. Ready-to-eat cereals, puffed and uncoated	4	0	0	0
C3. Ready-to-eat cereals, puffed and coated without fruit or nuts	88	0	24 (27.3%)	8 (9.1%)
C4. Ready-to-eat cereals, fruit and nut, granola type	169	19 (11.2%)	24 (14.2%)	0
C5. Bran and wheat germ, milled flax, etc.	38	5 (13.2%)	0	0
C6. Flours and cornmeal	66	0	0	2 (3.0%)
C7. Grains	279	2 (0.7%)	0	82 (29.4%)
C8. Pastas	492	21 (4.3%)	0	32 (6.5%)
C10. Starch	10	0	0	1 (9.1%)
C11. Stuffing	118	0	0	9 (90.0%)
**Category C Total**	**1,275**	**48 (3.8%)**	**53 (4.2%)**	**134 (10.5%)**
**D. Dairy Products & Substitutes**				
D1. Cheese	568	126 (22.2%)	1 (0.2%)	48 (8.5%)
D2. Cottage cheese	21	1 (4.8%)	1 (4.8%)	0
D3. Cheese used as ingredient	14	7 (50.0%)	0	0
D4. Hard cheese	44	15 (34.1%)	0	6 (13.6%)
D5. Quark	110	45 (40.9%)	5 (4.5%)	30 (27.3%)
D6. Cream and cream substitutes	41	0	0	0
D7. Powder cream and cream substitutes	9	2 (22.2%)	0	0

TRA Category*	n	'High in' nutrient-of-concern type, n(%) Saturated fat	Sugars (total)	Sodium
D8. Aerosol/whipped cream and cream substitutes	22	9 (40.9%)	1 (4.5%)	0
D10. Evaporated/condensed milk	17	0	7 (41.2%)	0
D11. Milk, buttermilk, milk-based drinks, plant-based milk substitutes	200	16 (8.0%)	44 (22.0%)	7 (3.5%)
D12. Fermented dairy drinks	59	1 (1.7%)	47 (79.7%)	1 (1.7%)
D13. Shakes and smoothies	25	7 (28.0%)	18 (72.0%)	1 (4.0%)
D14. Sour cream	24	13 (54.2%)	0	0
D15. Yogurt	338	54 (16.0%)	240 (71.0%)	0
**Category D Total**	**1,492**	**296 (19.8%)**	**364 (24.4%)**	**93 (6.2%)**
**E. Desserts**				
E1. Ice cream, frozen yogurt, sherbet, etc. in tubs	277	224 (80.9%)	273 (98.6%)	2 (0.7%)
E2 Ice cream, frozen yogurt, sherbet, etc. as cakes, cones	61	49 (80.3%)	52 (85.2%)	1 (1.6%)
E3 Ice cream, frozen yogurt, sherbet, etc. as pops, bars	153	81 (52.9%)	84 (54.9%)	0
E4. Sundaes	8	8 (100.0%)	8 (100.0%)	0
E5. Custard, gelatin, pudding	180	20 (11.1%)	134 (74.4%)	68 (37.8%)
**Category E Total**	**679**	**382 (56.3%)**	**551 (81.1%)**	**71 (10.5%)**
**F. Dessert Toppings & Fillings**				
F1. Dessert toppings	29	4 (13.8%)	26 (89.7%)	0
F2. Cake frostings‡	35	4 (11.4%)	35 (100.0%)	0
F3. Pie fillings	30	0	26 (86.7%)	1 (3.3%)
**Category F Total**	**94**	**8 (8.5%)**	**87 (92.5%)**	**1 (1.1%)**
**G. Eggs & Substitutes**				
G1. Egg mixtures	5	0	0	2 (40.0%)
G2. Eggs	56	0	0	2 (3.6%)
**Category G Total**	**61**	**0**	**0**	**4 (6.6%)**
**H. Fats & Oils**				
H1. Butter, margarine, lard, etc.	112	0	0	0
H2. Vegetable oil	166	0	0	0
H4. Dressings for salad	283	39 (13.8%)	4 (1.4%)	145 (51.2%)
H5. Mayonnaise and mayonnaise-type dressing	68	0	0	0
H6. Spray oil	23	0	0	0
**Category H Total**	**652**	**39 (6.0%)**	**4 (0.6%)**	**145 (22.2%)**
**I. Seafood & Substitutes**				
I1. Anchovies, caviar	9	0	0	6 (66.7%)
I2. Marine and freshwater animals with sauce	50	9 (18.0%)	3 (6.0%)	42 (84.0%)
I3. Marine and freshwater animals without sauce	191	16 (8.4%)	0	106 (55.5%)
I4. Canned marine and freshwater animals	147	0	0	27 (18.4%)
I5. Smoked/pickled marine and freshwater animals	49	3 (6.1%)	0	30 (61.2%)
**Category I Total**	**446**	28 (6.3%)	3 (0.7%)	211 (47.3%)
**J. Fruits & Fruit Juices**				
J1. Fruits (fresh, frozen, canned, coated, and uncoated)	186	1 (0.5%)	87 (46.8%)	0
J2. Berries	15	0	0	0
J3. Melons	5	0	0	0
J4. Avocados	1	0	0	0
J5. Cranberries, lemons, limes	3	0	0	0
J6. Fruit sauces and purees	65	0	19 (29.2%)	0
J7. Dried fruits	131	3 (2.3%)	47 (35.9%)	0
J8. Candied/pickled fruits	21	0	12 (57.1%)	9 (42.9%)
J9. Fruits for garnish	5	0	0	0
J11. Juices, nectars, fruit drinks	603	3 (0.5%)	547 (90.7%)	2 (0.3%)
J12. Fruit juices used as ingredients	10	0	0	0
**Category J Total**	**1,045**	**7 (0.7%)**	**712 (68.1%)**	**11 (1.1%)**

TRA Category[*]	n	'High in' nutrient-of-concern type, n(%) Saturated fat	Sugars (total)	Sodium
**K. Legumes**				
K1. Tofu or tempeh	23	0	5 (21.7%)	1 (4.3%)
K2. Beans, lentils, etc.	164	0	0	18 (11.0%)
**Category K Total**	**187**	**0**	**5 (2.7%)**	**19 (10.2%)**
**L. Meats & Substitutes**				
L1. Pork rinds and bacon	41	38 (92.7%)	0	19 (46.3%)
L2. Beef, pork and poultry breakfast strips	6	1 (16.7%)	0	4 (66.7%)
L3. Dried meat and poultry	96	73 (76.0%)	8 (8.3%)	95 (99.0%)
L4. Luncheon meats	85	30 (35.3%)	0	73 (85.9%)
L5. Sausage products	160	117 (73.1%)	0	153 (95.6%)
L6. Cust of meat & poultry without sauce	125	45 (36.0%)	1 (0.8%)	89 (71.2%)
L7. Patties, ground meat with and without breading	214	92 (43.0%)	0	137 (64.0%)
L8. Cured meats	86	15 (17.4%)	0	83 (96.5%)
L9. Canned meats	27	16 (59.3%)	0	20 (74.1%)
L10. Meat and poultry with sauce	112	61 (54.5%)	35 (31.3%)	109 (97.3%)
**Category L Total**	**952**	**488 (51.3%)**	**44 (4.6%)**	**782 (82.1%)**
**M. Miscellaneous**				
M1. Baking powder, baking soda, yeast[†]	25	0	2 (8.0%)	1 (4.0%)
M2. Baking decoration	20	0	0	0
M3. Bread crumbs	241	23 (9.5%)	134 (55.6%)	141 (58.5%)
M5. Cocoa powder	5	0	0	0
M7. Chewing gum	23	0	0	0
M8. Salad and potato toppers	166	2 (8.7%)	0	0
M9. Salt, salt substitutes[†]	36	0	0	78 (47.0%)
M10. Spices and herbs without salt	19	0	0	0
M11. Coconut milk	14	19 (100.0%)	0	0
M12. Dried coconut	25	14 (100.0%)	0	0
**Category M Total**	**552**	**58 (10.5%)**	**136 (24.6%)**	**220 (39.9%)**
**N. Combination Dishes**				
N1. Combination dishes	529	135 (25.5%)	47 (8.9%)	400 (75.6%)
N2. Burritos, pizzas, sandwiches, meat pie, etc. [†]	408	220 (53.9%)	3 (0.7%)	329 (80.6%)
N3. Hors d'oeuvres	124	55 (44.4%)	5 (4.0%)	95 (76.6%)
**Category N Total**	**1,061**	410 (38.6%)	55 (5.2%)	824 (77.7%)
**O. Nuts & Seeds**				
O1. Nuts and seeds (not used for snacks) [‡]	140	0	0	0
O2. Nut pastes and creams	7	3 (42.9%)	7 (100.0%)	0
O3. Nut butters	101	12 (11.9%)	1 (1.0%)	1 (1.0%)
O4. Nut flours	4	2 (50.0%)	0	0
**Category O Total**	**252**	17 (6.7%)	8 (3.2%)	1 (0.4%)
**P. Potatoes**				
P1. French fries	65	1 (1.5%)	0	12 (18.5%)
P2. Mashed, stuffed, candied potatoes	37	18 (48.6%)	0	30 (81.1%)
P3. Fresh, canned, frozen potatoes	29	0	1 (3.4%)	4 (13.8%)
**Category P Total**	**131**	**19 (14.5%)**	**1 (0.8%)**	**46 (35.1%)**
**Q. Salads**				
Q1. Salads	84	23 (27.4%)	9 (10.7%)	42 (50.0%)
Q3. Pasta, potato or grain-based salad	20	6 (30.0%)	0	14 (70.0%)
**Category Q Total**	**104**	**29 (27.9%)**	**9 (8.7%)**	**56 (53.8%)**
**R. Sauces & Dips**				
R1. Dipping sauces	158	7 (4.4%)	70 (44.3%)	94 (59.5%)
R2. Dips and spreads	183	30 (16.4%)	5 (2.7%)	30 (16.4%)
R3. Major main entrée sauce	225	44 (19.6%)	12 (5.3%)	167 (74.2%)
R4. Minor main entrée sauce	264	36 (13.6%)	33 (12.5%)	138 (52.3%)
R5. Major condiments[†,‡]	293	0	12 (4.1%)	127 (43.3%)
R6. Minor condiments[†,‡]	121	0	0	1 (0.8%)
**Category R Total**	**1,244**	**117 (9.4%)**	**132 (10.6%)**	**557 (44.8%)**

TRA Category*	n	'High in' nutrient-of-concern type, n(%)		
		Saturated fat	Sugars (total)	Sodium
**S. Snacks**				
S1. Chips, pretzels, etc.†	562	116 (20.6%)	33 (5.9%)	201 (35.8%)
S2. Nuts or seeds (used as snacks)	252	44 (17.5%)	48 (19.0%)	13 (5.2%)
S3. Meat or poultry sticks	31	26 (83.9%)	0	28 (90.3%)
**Category S Total**	**845**	**186 (22.0%)**	**81 (9.6%)**	**242 (28.6%)**
**T. Soups**				
T1. All varieties of soups (includes broth)	475	111 (23.4%)	42 (8.8%)	447 (94.1%)
**Category T Total**	**475**	**111 (23.4%)**	**42 (8.8%)**	**447 (94.1%)**
**U. Sugars & Sweets**				
U1. Candies, confectionaries, chocolates	534	364 (68.2%)	449 (84.1%)	0
U3. Hard candies	20	0	7 (35.0%)	0
U4. Baking candies	47	47 (100.0%)	10 (21.3%)	0
U5. Breath mints	2	0	0	0
U7. Confectioner's or icing sugar	3	0	0	0
U8. Honey, molasses, bread spreads	72	8 (11.1%)	9 (12.5%)	0
U9. Jams, jellies, fruit spreads	225	0	23 (10.2%)	0
U10. Fruit leather	20	1 (5.0%)	20 (100.0%)	0
U11. Marshmallows	12	0	12 (100.0%)	0
U12. Sugars†,‡	45	0	0	0
U14. Syrups used as toppings	52	0	0	0
U15. Syrups used as ingredients	20	0	2 (10.0%)	0
**Category U Total**	**1,052**	**420 (39.9%)**	**532 (50.6%)**	**0**
**V. Vegetables**				
V1. Vegetables without sauce	407	0	0	25 (6.1%)
V2. Vegetables with sauce	10	5 (50.0%)	0	6 (60.0%)
V3. Vegetables used for garnishing/flavouring†,‡	27	0	0	0
V4. Chili pepper & green onion†	42	1 (2.4%)	0	24 (57.1%)
V5. Seaweed, dehydrated mushrooms	19	0	0	0
V6. Sprouts	1	0	0	0
V7. Vegetable juice and drink	58	0	9 (15.5%)	18 (31.0%)
V8. Olives	65	2 (3.1%)	0	40 (61.5%)
V9. Sun-dried tomatoes and other pickled or oil-packed vegetables	170	0	1 (0.6%)	109 (64.1%)
V10. Relish	15	0	0	1 (6.7%)
V11. Vegetable paste	12	0	0	0
V12. Vegetable sauce or purée	34	0	0	5 (14.7%)
**Category V Total**	**860**	**8 (0.9%)**	**10 (1.2%)**	**228 (26.5%)**
**W. Foods for <4 years old⁂**				
W1. Cereals to be prepared†	41	0	20 (48.8%)	0
W2. Ready-to-eat cereals and cereal bars†	9	0	9 (100.0%)	0
W3. Cookies, biscuits, etc. †	46	0	6 (13.0%)	0
W4. Strained meat, desserts, combination dishes†	89	0	60 (67.4%)	0
W5. Combination dishes†	8	0	0	0
W6. Juices†	2	0	2 (100.0%)	0
**Category W Total**	**195**	**0**	**97 (49.7%)**	**0**
**OVERALL TOTAL**	**17,008**	**3,563 (20.9%)**	**4,055 (23.8%)**	**4,543 (26.7%)**

All values are presented as n (%). Pre-packaged foods in Food Label Information and Price (FLIP) 2017 were used (n=17,008). FOPL regulations mandate pre-packaged foods to display a 'High in' nutrition symbol if they meet and/or exceed thresholds for nutrients-of-concern (saturated fat, total sugars, or sodium) [1]. *Health Canada's Table of Reference Amounts for Food (TRA) [2] was used to define food categories. †Indicates categories with products that were missing values for saturated fat (n=217; 1.3% overall). ‡Indicates categories with products that were missing values for sugars (n=5; 0.03% overall). ‡Indicates categories with products that were missing values for sodium (n=10; 0.06% overall). ⁂Although foods for <1-year-olds would be exempted from FOPL regulations [1], all foods for <4-year-olds with a Nutrition Facts table were included as only the minimum age for consumption (e.g., ≥ 6-month-olds), not maximum age for consumption, are indicated in these foods. Abbreviations: FLIP, Food Label Information and Price; FOPL, front-of-pack labelling; TRA, Table of Reference Amounts for Food.

Assessing the extent to which front-of-pack labelling regulations could support healthy eating among Canadians

Lee JJ, Mulligan C, Jeong H, L'Abbe MR

References:

1. Lee JJ, Mulligan C, L'Abbé MR. Development and validity testing of the Canadian Food Scoring System (CFSS), a nutrient profile model based on the recommendations of Canada's food guide 2019. Appl Physiol Nutr Metab. 2024. doi: 10.1139/apnm-2024-0034 %M 39013203.

2. Health Canada. Table of Reference Amounts for Food. 2016 [cited 2019 July 15]. Available from: https://www.canada.ca/en/health-canada/services/technical-documents-labelling-requirements/table-reference-amount-food-2016.html.
